# Heavy Metal Contaminations in Herbal Medicines: Determination, Comprehensive Risk Assessments, and Solutions

**DOI:** 10.3389/fphar.2020.595335

**Published:** 2021-01-14

**Authors:** Lu Luo, Bo Wang, Jingwen Jiang, Martin Fitzgerald, Qin Huang, Zheng Yu, Hui Li, Jiqing Zhang, Jianhe Wei, Chenyuyan Yang, Hui Zhang, Linlin Dong, Shilin Chen

**Affiliations:** ^1^ Institute of Chinese Materia Medica, China Academy of Chinese Medical Sciences, Beijing, China; ^2^ Hubei Institute for Drug Control, Wuhan, China; ^3^ West China Biomedical Big Data Center, West China Hospital, Sichuan University, Chengdu, China; ^4^ Department of Life Sciences, University of Westminster, London, United Kingdom; ^5^ College of Medical Information and Engineering, Chengdu University of Traditional Chinese Medicine, Chengdu, China; ^6^ Institute of Botany, Chinese Academy of Sciences, Beijing, China; ^7^ Hainan Provincial Key Laboratory of Resources Conservation and Development of Southern Medicine, Hainan Branch of the Institute of Medicinal Plant Development, Chinese Academy of Medical Sciences and Peking Union Medical College, Haikou, China; ^8^ Institute of Medical Information, Chinese Academy of Medical Sciences and Peking Union Medical College, Beijing, China; ^9^ Akupunktur Akademiet, Hovedgaard, Denmark

**Keywords:** heavy metal, risk assessment, safety and quality, herbal mecidine, extrinsic contamination

## Abstract

Heavy metal contamination in herbal medicines is a global threat to human beings especially at levels above known threshold concentrations. The concentrations of five heavy metals cadmium (Cd), lead (Pb), arsenic (As), mercury (Hg) and copper (Cu) were investigated using Inductively Coupled Plasma Optical Mass Spectrometry (ICP-MS) with 1773 samples around the world. According to Chinese Pharmacopoeia, 30.51% (541) samples were detected with at least one over-limit metal. The over-limit ratio for Pb was 5.75% (102), Cd at 4.96% (88), As at 4.17% (74), Hg at 3.78% (67), and of Cu, 1.75% (31). For exposure assessment, Pb, Cd, As, and Hg have resulted in higher than acceptable risks in 25 kinds of herbs. The maximal Estimated Daily Intake of Pb in seven herbs, of Cd in five, of Hg in four, and As in three exceeded their corresponding Provisional Tolerable Daily Intakes. In total 25 kinds of herbs present an unacceptable risk as assessed with the Hazard Quotient or Hazard Index. Additionally, the carcinogenic risks were all under acceptable limits. Notably, As posed the highest risk in all indicators including Estimated Daily Intake, Hazard Index, and carcinogenic risks. Therefore further study on enrichment effect of different states of As and special attention to monitoring shall be placed on As related contamination.

## Introduction

Having been utilized as traditional folk remedies for thousands of years (Maiga et al., 2005), medicinal plants gained an increasingly important role in the pharmaceutical, health food, and natural cosmetic industries (Kim et al., 2016). It was reported that a total of 60,107 COVID-19 cases (85.20% of the total cases) in China were treated by Chinese herbal medicines with positive results in all infection stages, including significant symptom management, lower rates of deterioration and mortality, faster recovery as well as disease prevention on February 17, 2020 (Ministry of Science and Technology, 2020). However, concerns grew regarding the safety of herbal medicines after studies indicated that high levels of heavy metals were present in some herbal medicines. This was a problem more frequently encountered in traditional medical therapy, considered to be a cause of several health disorders (Maiga et al., 2005; Brookes et al., 2019). Samples taken from both developed and developing countries have shown high levels of potentially toxic heavy metals in products available to the public (Awodele et al., 2013). It is known that numerous traditional medicines can give rise to severe adverse renal pathology, the mechanism of which is yet not fully certain but has been associated with heavy metal toxicity (Awodele et al., 2013). Uptake of heavy metals by plants and subsequent accumulation along the food chain is a potential threat to animal and human health (Jiwan and Kalamdhad, 2011). Particularly as heavy metals are known to have low renal excretion rates, potentially resulting adverse effects in humans even at very low concentrations (Jamroz et al., 2015). They are not easily metabolized by body and are found to accumulate in the soft tissues (Jiwan and Kalamdhad, 2011). They produce toxic effects due to their interference in many known normal biochemical and metabolic processes (Kurban et al., 2015). Several health problems were linked to excessive uptake of dietary heavy metals, including decreased immunity, cardiac dysfunction, fetal malformation, impaired psychosocial and neurological behavior (Harris et al., 2011). Pb and Cd are not essential elements that are required neither in the human body nor in plants, and which cause various bimolecular adverse functional effects at low level doses (Maiga et al., 2005). Though an essential component of many enzymes, excessive intake of Cu can cause dermatitis, irritation of the upper respiratory tract, abdominal pain, nausea, diarrhea, vomiting, and liver damage (Harris et al., 2011). While As and Hg can damage pulmonary, nervous, renal and respiratory systems, as well as causing skin pathology (Jarup, 2003; Mahurpawar, 2015). It may also induce disorders in the central nervous system, liver, lungs, heart, kidney and brain. Leading to hypertension, abdominal pain, skin eruptions, intestinal ulcer and is associated with various types of cancers (Paulsen et al., 2012). It is therefore necessary and a matter of urgency to conduct a comprehensive risk assessment of heavy metal contamination in herbal medicines.

To explore and guarantee the safety of herbal medicines, multiple studies regarding heavy metal contamination in herbal medicines have been carried out in China (Ye et al., 2013; Li et al., 2018), India (Singh et al., 2016), Iran (Behnam et al., 2017), Egypt (Abou and Abou Donia, 2000), South Africa (Mulaudzi et al., 2017), United States (Harris et al., 2011), Brazil (Leal et al., 2013), and Australia (Kim et al., 2016), etc. Though a number of studies have been conducted regarding heavy metal contamination in herbal plants, most were with limited sample numbers and categories. Given the severe consequence it may bring to health and environment, exposure risk assessment (Kirkham et al., 2010; Okatch et al., 2012), hazard quotient (Li et al., 2018), and ecological risk assessment (Awodele et al., 2013), have been conducted by researchers, showing that heavy metal contamination in herbal medicines is an area requiring immediate attention, with potential risk to human health having now being demonstrated (Sandilyan and Kathiresan, 2014). Though studies now show that exposure of heavy metals through general dietary consumption contribute negatively to human health (Wei and Cen, 2020), very few have conducted comprehensive health risk assessments, with large sample numbers focusing on herbal medicines. Furthermore, specific identification of metals is required for accurate diagnosis due to considerable overlap between various clinical syndromes associated with heavy metal poisoning (Kurban et al., 2015). Therefore a study of accurately quantified heavy metal contents in herbal medicines appears necessary to further assess and justify of the dosage of herbal formulas. This study assesses contamination levels and the health risk to humans posed by heavy metals more specifically in herbal medicines, providing an evidence base on which to further build prevention measures, establish relative standards, and control external contamination. Through investigation and suggested recommendations in able to significantly reduce or eliminate the levels of heavy metals in herbal medicines.

## Materials and Methods

### Sample Collection and Detection

A total of 1773 crude plant extract samples from 2014 to 2019, representing 86 different kinds of commonly used herbal medicines were collected for examination of heavy metals. As a part of a heavy metal detection project for Chinese Pharmacopoeia (2020 edition) (National Pharmacopoeia Commission, 2020) and based on the principle that only herbal medicines from large-scale production areas would be considered, at least three samples for each herbal medicine were collected from one to 13 different sampling locations, which were purchased in four major Chinese herbal markets (Anhui, Henan, Chengdu, Hebei). Sampling locations were chosen according to areas of traditional production of the herbal medicines, without regard to possible pollution sources. Each bulk sample was harvested, cleaned, and processed according to the method required by Chinese Pharmacopoeia (National Pharmacopoeia Commission, 2020). Precisely 0.5 g sample (Electronic balance, Mettler Toledo) was first ground into powder, then soaked in polytetra fluoroethylene (PTFE) tank with 6 mL nitric acid (HNO_3_) added overnight. It was then predigested for 1 h on a temperature controlled electronic hotplate with 1 mL hydrogen peroxide solution (H_2_O_2_) added. After cooling down, more HNO_3_ was added to up to volume of 7 mL. The amount of each metal was added before microwave digestion. The PTFE tank was then placed in Multiwave PRO Microwave Digestion Apparatus (Anton Paar) for further digestion before being placed on a 130°C electronic hotplate, until reduced to 1 mL. Microwave digestion was set on a digestion program. For the first stage, 1100 W, maintained for 10 min then further, for the second stage, 1,400 W for 15 min and held for 20 min (Gonzalez-Martin et al., 2018). The detailed methodology is listed in the appendix (Supplementary Table S1). The tank was then removed for cooling and 50 mL of the digested liquid transferred to a graduated flask. The tank was flushed with low volume amounts of water multiple times and the complete washings added to the graduated flask, 200 μL of single-element standard solution (1 mg L^−1^) was added. Then diluted up to the mark of the graduated flask with water, shaken and set aside. The blank solution was prepared in the same way except that no standard solution of single element and sample powder were added (Pohl, 2016).

The resulting solution was analyzed using a Scientific X Series Inductively-Coupled Plasma Mass Spectrometer (ICP-MS) (Thermo Fisher Scientific, Waltham, MA). Heavy metal results from the ICP-MS were quantified against standard curves generated from 1 blank and at least 4 standard reference solutions (High-Purity Standards, Charleston, SC) run separately. Quality control was assessed by running a laboratory reagent blank after every 10 samples. The detection limit was based on consideration of the blank runs, concentration of the low standard in the calibration curve and the sample preparation procedure. Based on this method, the limit of detection was considered equivalent to the limit of quantification (Wang and Hansen, 2004; Pereira et al., 2010). The blank solution was run for 14 times and the first three were discarded considering they were not stable. The limit of detection (LOD) was automatically calculated with standard deviation of the remaining 11 runs, divided by the slope of the standard curve. The LOD achieved for each metal was 0.1 mg kg^−1^ for Cu, 0.01 mg kg^−1^ for As, 0.005 mg kg^−1^ for Cd, 0.001 mg kg^−1^ for Hg, and 0.01 mg kg^−1^ for Pb. Detailed parameters could be found in supplementary materials (Supplementary Tables S1.1–S1.23).

In our experiment, standard solutions of Cu (GSB04-1725, 1,000 mg L^−1^), As (GSB04-1714, 1,000 mg L^−1^), Cd (GSB04-1721, 1,000 mg L^−1^), Hg (GSB04-1729, 1,000 mg·L), Pb (GSB04-1742, 1,000 mg L^−1^) were purchased from the National Nonferrous Metals and Electronic Materials Analysis and Testing Center, while Analytical Reagents (AR) and nitric acid (HNO_3_) were purchased from Merck Co., Ltd., and guaranteed reagents (GR) hydrogen peroxide and hydrochloric acid from Sinopharm Chemical Reagent Co., Ltd. Each single-element standard solution was measured precisely and then diluted with 5% HNO_3_ to make a mixed solution containing 1 μg mL^−1^. For a reference stock solution, single-element standard solutions of the five metals were separately taken and diluted with 5% nitric acid to make solutions containing 5 μg of Pb and As, 50 μg of Cu, 2.5 μg of Cd, and 0.5 μg of Hg. For preparation of a reference standard curve, precise measurements were taken of the above stock solution diluted with 5% nitric acid to make standard mixtures (ng·mL^−1^): 0, 1, 5, 10, 20, and 50 ng mL^−1^ As or Pb; 0, 10, 50, 100, 200, and 500 ng mL^−1^ Cu; 0, 0.5, 2.5, 5, 10, and 25 ng mL^−1^ Cd; 0, 0.1, 0.5, 1, 2, and 5 ng mL^−1^ Hg (Harris et al., 2011).

The conditions for Inductively coupled plasma mass spectrometry (ICP-MS) conditions were: Radio Frequency (RF) power: 1400 W; sampling depth: 15 mm; auxiliary gas (argon) flow rate: 0.8 L min^−1^; cooling gas flow rate: 13.0 L min^−1^; peristaltic pump speed: 30.0 L min^−1^; channel three; repeat for three times; scan for 100 times, automatic detection. Before samples were measured, the instrument was optimized to perform under the optimal conditions.

### Quantification of Heavy Metal Contamination

Mean concentrations, general detection rate, and detection rates of each metal were calculated. Figures were plotted using *R* language [no IDE (integrated development environment), *R* from the linux terminal *R* version 3.5.1. (2018-007-02)--“Feather Spray” Copyright (C) 2018, the *R* Function for Statistic Computing Platform: x86_64-pc-linux-gnu (64-bit)] (Wang et al., 2009; The R core team, 2020).

### Over-limit Ratio of Five Heavy Metals

In total, 27 currently available permissible limits containing five heavy metals of herbal medicines were obtained from 20 countries or regions and seven international organizations (Supplementary Table S2). The detailed calculation of both general over-limit ratio and that of each metal in different producing areas were shown in appendix (Supplementary Table S3). Numbers of over-limit samples and metals were calculated from within across five medicinal herbal properties, including: flos (flower), folium and cortex (leaf and bark), fructus and semen (fruit and seed), herba and others (the whole plant), and radix and rhizoma (root and rootstock).

$$\text{Over}‐\text{Limit ratio}=\left(C_{\mathrm{detected}}-{\mathrm{Limit}}_{\mathrm{metal}}\right)\times 100\%$$ (1)

$$\text{Times Over Permissible Limits}=\frac{C_{\mathrm{detected}}-{\mathrm{Limit}}_{\mathrm{metal}}}{{\mathrm{Limit}}_{\mathrm{metal}}}\times 100\%$$ (2)

### The Health Risk Assessments of Heavy Metal Contamination in Herbal Medicines

Exposure assessment (EDI), non-carcinogenic risk assessment (HQ, HI), and carcinogenic risk assessment (CR) were employed to explore the potential health impacts from heavy metal contamination in herbal medicines. The U.S. Environmental Protection Agency (EPA) has replaced the Tolerable daily intake (TDI) with RfD, which is defined as “an estimate (with uncertainty spanning perhaps an order of magnitude) of a daily exposure for the human population (including sensitive subgroups) that is likely to be without an appreciable risk of deleterious effects during a lifetime.” Use of the term RfD is intended to avoid any implication that exposure to the chemical is completely “safe” or “acceptable” (Peter et al., 2015; Gunnar et al., 2019). We adopted provisional tolerable daily intake (PTDI) in exposure assessment, to compare the estimated daily intake (EDI) with PTDI. While for non-carcinogenic risk assessment, we adopted the Oral reference dose (RfD), to evaluate it by comparing an exposure level over a specified time period (eg, lifetime) with a reference dose derived for a similar exposure period (Sawut et al., 2018; USEPA, 1989; USEPA 2009; USEPA 2012). Additionally, the minimal, mean and maximal concentrations of each metal in each herbal medicines were applied for calculations with the equations below:

### Exposure Assessment

$$\text{EDI}=\frac{C\times \text{IRD}}{\text{BW}}$$ (3)

The estimated daily intake (EDI, mg kg^−1^ day^−1^·bw) of each metal in each sample was calculated, before comparison with its corresponding provisional tolerable daily intake (PTDI). The PTDIs (mg·kg^−1^·d^−1^) of As, Cu, Hg, Pb, and Cd are 0.00214, 0.5, 0.00057, 0.00357, and 0.00083, respectively (Sawut et al., 2018). *C* (mg·kg^−1^) in the equation here refers to the concentrations detected of each metal in herbal medicines (maximal, mean and minimal concentrations were all considered). IRD (kg·day^−1^) refers to daily ingestion rate, which signifies the daily dosage of herbal medicines. Here the maximal dosage specified in CP (2020 edition) was applied. BW (kg) is body weight, and average human weight of 60 kg was applied in the equation (Zhao et al., 2010).

### Non-carcinogenic Risk Assessment

The non-cancer risk was evaluated by comparing an exposure level over a specified time period (eg, lifetime) with a reference dose derived for a similar exposure period. The non-cancer risk can be characterized as a hazard quotient (HQ) (USEPA, 1989; USEPA 2009; USEPA 2012; Sawut et al., 2018).

$$\text{HQ}=\frac{\text{C}\times \text{IR}\times \text{Ef}\times \text{Ed}\times \text{t}}{\text{AT}\times \text{BW}\times \text{RfD}}$$ (4)

IR (kg·day^−1^) is the daily dosage of herbal medicine, and according to a questionnaire on herbal medicine consumption of 20917 people, the 95th percentile of daily dosage of general herbal medicine consumption is 0.5 kg; Ef (day) is exposure frequency, here the 95th percentile of annual consumption on herbal medicine was adopted which was set 90 days per year; Ed (year) is the exposed days over a lifetime which was set as 20 years; AT (day) is the average lifetime = 365 days × 70 years, while t is the transfer rate of heavy metal to herbal detection, which is 14% for Cd, Cu, and Pb, 35% for As, and 24% for Hg; The transfer rates were referred from the investigation conducted by Zuo et al. (2019), which were estimated based on several fine quantification researches on transfer rates of heavy metals in herbal medicines conducted in China (Wang et al., 2014; Gan et al., 2016; Jin et al., 2017; Pan et al., 2017; Zuo et al., 2017). RfD refers to Oral reference dose (mg^−1^·kg^−1^·day), which is 0.0035 for Pb, 0.0005 for Cd, 0.0003 for As, 0.0003 for Hg and 0.04 for Cu (Li et al., 2019).

HQs of five heavy metals in each herbal medicine were summed up to obtain non-carcinogenic Hazard Index (HI). If HQ or HI is less than 1, there will not be obvious risk for exposed population from metal exposure in herbal medicine. If HQ or HI is equal to or above 1, the risk will be considered unacceptable. As the HQ or HI increases, the risk also does. The contributions of HQs of each metal (HQ_m_) to the total HI were calculated to explore which metal contributed the most serious risks (Farmer et al., 2019).

HI=∑HQ (5)

$${\text{Contribution of HQ}}_{\text{m}}\text{ to HI}=\left({\mathrm{HQ}}_{m}-\mathrm{HI}\right)\times 100\%$$ (6)

### Carcinogenic Risk Assessment

$$\text{CR}=\frac{C\times \mathrm{IR}\times \mathrm{Ef}\times \mathrm{Ed}\times t\times \mathrm{SFo}}{\mathrm{AT}\times \mathrm{BW}}$$ (7)

SFo (mg·kg^−1^·day^−1^·bw) is the Oral Slope Factor signifying cancer severity, only three heavy metals were proven with certain SFs: 6.1 for Cd, 1.5 for As and 0.0085 for Pb, while 10^-6^ is the conversion factor (USEPA, 1989; USEPA 2009; USEPA 2012; Liu et al., 2013; Farmer et al., 2019). CR of these three metals in the same herbal medicine was also summed up to give total CR of single herbal medicine. If CR is higher than 10^−6^, which means one case of cancer over one million exposed people, it is considered unacceptable (Fakhari et al., 2017; Onyele and Anyanwu, 2018).

## Results

### The Content of Five Heavy Metals in 1773 Herbal Medicines

**FIGURE 1:**
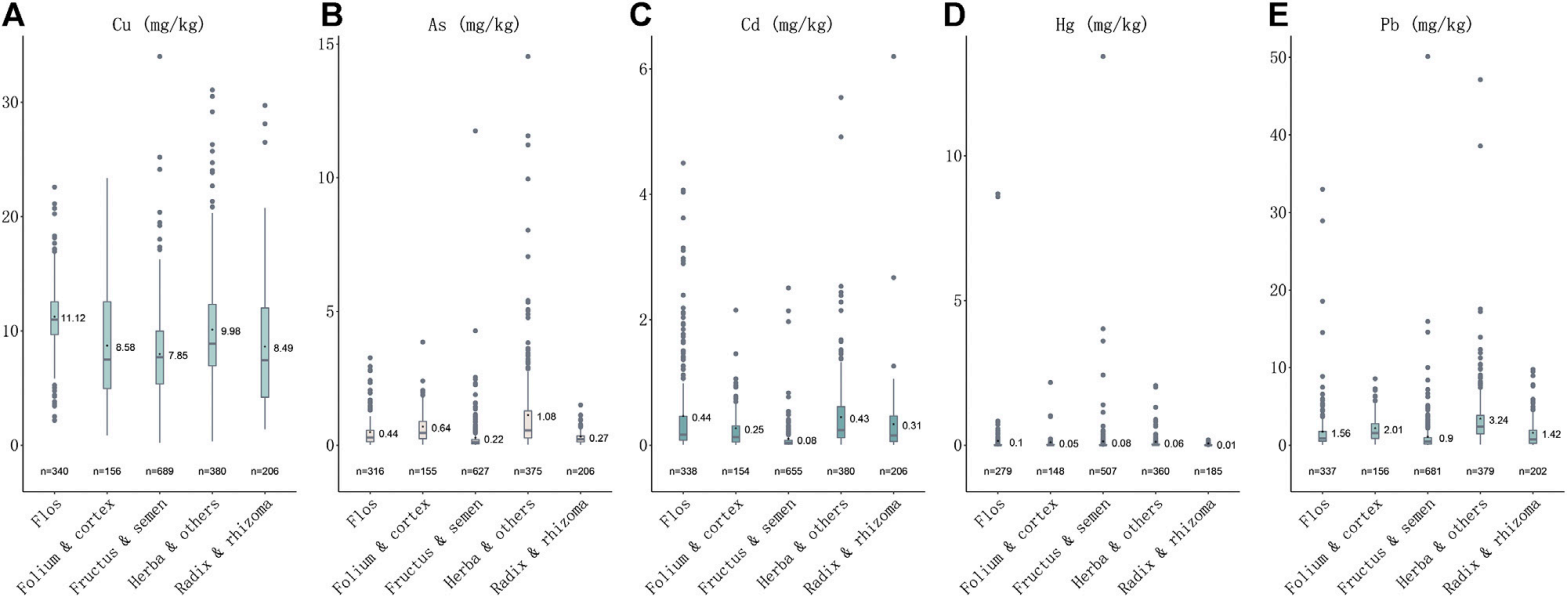
Plotbox showing concentrations of five heavy metals in five medicinal properties. **(A)** Concentrations of Cu in five medicinal properties. **(B)** Concentrations of As in five medicinal properties. **(C)** Concentrations of Cd in five medicinal properties. **(D)** Concentrations of Hg in five medicinal properties. **(E)** Concentrations of Pb in five medicinal properties.

Heavy metals were detected in all 1773 samples. The order of detection rates of heavy metal concentrations in herbal medicines is Cu (1771, 99.89%) > Pb (1755, 98.98%) > Cd (173, 97.74%) >As (1,679, 94.70%) > Hg (1,497, 84.43%). The contents (mg·kg^−1^) of each medicinal property were also calculated, the highest content of Cu, Cd and Hg was 11.12 ± 2.73, 0.439 ± 0.686 and 0.081 ± 0.667, respectively, in Flos (*n* = 340); the highest content of As and Pb was 1.06 ± 1.56 and 3.23 ± 3.83, in Herba and others (*n* = 380) (Table 1). The orders of mean concentrations detected of five heavy metals in five plant properties are: for Cu, flos > herba and others > folium and cortex > radix and rhizoma > fructus and semen; for As, herba and others > folium and cortex > flos > radix and rhizoma > fructus and semen; for Cd, flos > herba and others > radix and rhizoma > folium and cortex > fructus and semen; for Hg, flos > fructus and semen > herba and others > folium and cortex > radix and rhizoma; while for Pb, herba and others > folium and cortex > flos > radix and rhizoma > fructus and semen (Figure 1). The highest concentration detected for Cu was in herbal medicine *Schisandra chinensis* (Turcz.) Baill. (34.01 mg kg^−1^), the highest concentration of As was in *Plantago asiatica* L. (14.53 mg kg^−1^), of Cd was in *Curcuma longa* L. (6.20 mg kg^−1^), of Hg was in *Chrysanthemum indicum* L. (8.69 mg kg^−1^), of Pb was in *Tetradium ruticarpum* (A.Juss.) T.G.Hartley (50.11 mg kg^−1^) (Table 1). In conclusion, all five heavy metals were widely detected in herbal medicines, particularly, Cu and Pb, most notably in flos and herba parts of medicinal plants.

**TABLE 1:** Health risk assessment scores of top risk-inducing herbal medicines.

Herbal medicine	Medicinal plant property	Max EDI (mg·kg^−1^·day^−1^·bw)				Max HQ				HI	Max CR			
		As	Cd	Hg	Pb	As	Cd	Hg	Pb		As	Cd	Pb	Total CR
*Mentha canadensis* L.	Herba and others					1.54				2.28				
*Plantago asiatica *L.	Herba and others	0.007			0.024	9.95			1.11	11.47	1.34E−06			
*Andrographis paniculata* (Burm.f.) Nees	Herba and others		0.001			5.51				7.02				2.18E−06
*Isatis tinctoria* L.	Folium and cortex					1.64				2.21				
*Pueraria montana* (Lour.) Merr.	Radix and rhizoma					1.03				1.35				
*Grona styracifolia* (Osbeck) H.Ohashi and K.Ohashi	Herba and others		0.001	0.001	0.008	1.79				3.47				
*Carthamus tinctorius* L.	Flos					2.02				2.41				
*Chrysanthemum indicum* L.	Flos			0.001		1.90		4.08		6.31				
*Tussilago farfara* L.	Flos					2.24		1.89		2.65				
*Forsythia suspensa* (Thunb.) Vahl	Fructus and semen			0.001		1.29				3.51				
*Ligustrum lucidum* W.T.Aiton	Fructus and semen					1.74				2.91				
*Taraxacum officinale* (L.) Weber ex F.H.Wigg.	Herba and others	0.002			0.004	6.82		`		8.62				
*Cornus officinalis* Siebold and Zucc.	Fructus and semen	0.002				8.05				8.66				
*Ziziphus jujuba* Mill.	Fructus and semen					1.58				1.83				
*Tetradium ruticarpum* (A.Juss.) T.G.Hartley	Fructus and semen			0.001	0.004	1.69		6.31	1.18	9.53			1.23E−07	
*Schisandra chinensis* (Turcz.) Baill.	Fructus and semen					1.07				1.49				
*Houttuynia cordata* Thunb.	Herba and others		0.002		0.004	1.97				3.42				
*Gardenia jasminoides* J.Ellis	Fructus and semen					2.93		1.14		4.57				
*Citrus × aurantium* L.	Fructus and semen					1.08				1.28				
*Perilla frutescens* (L.) Britton	Folium and cortex					2.64		1.02		4.02				
*Curcuma longa* L.	Radix and rhizoma		0.001				1.02			1.43		1.55E−06		
*Coptis chinensis* Franch.	Radix and rhizoma													
*Lonicera japonica* Thunb.	Flos				0.005					2.12				
*Chaenomeles lagenaria* (Loisel.) Koidz.	Fructus and semen									1.48				
*Lonicera confusa* DC.	Flos		0.001		0.004					1.81				

The estimated daily intakes (EDI, mg·kg^−1^·day^−1^·bw) above their corresponding provisional tolerable daily intakes (PTDI) were shown (The PTDIs (mg·kg^−1^·day^−1^) of As, Hg, Pb, and Cd are 0.00214, 0.00057, 0.00357, and 0.00083, respectively). The non-carcinogenic hazard quotient (HQ) and non-carcinogenic Hazard Index (HI) above one were shown. The carcinogenic risks (CR) higher than 10^-6^, which means one case of cancer over one million exposed people, is considered unacceptable, thus shown here. All scores in this table were calculated with maximal concentrations of each herbal medicine.

### Over-Limit Ratio of Five Heavy Metals in 1773 Herbal Medicines

**FIGURE 2:**
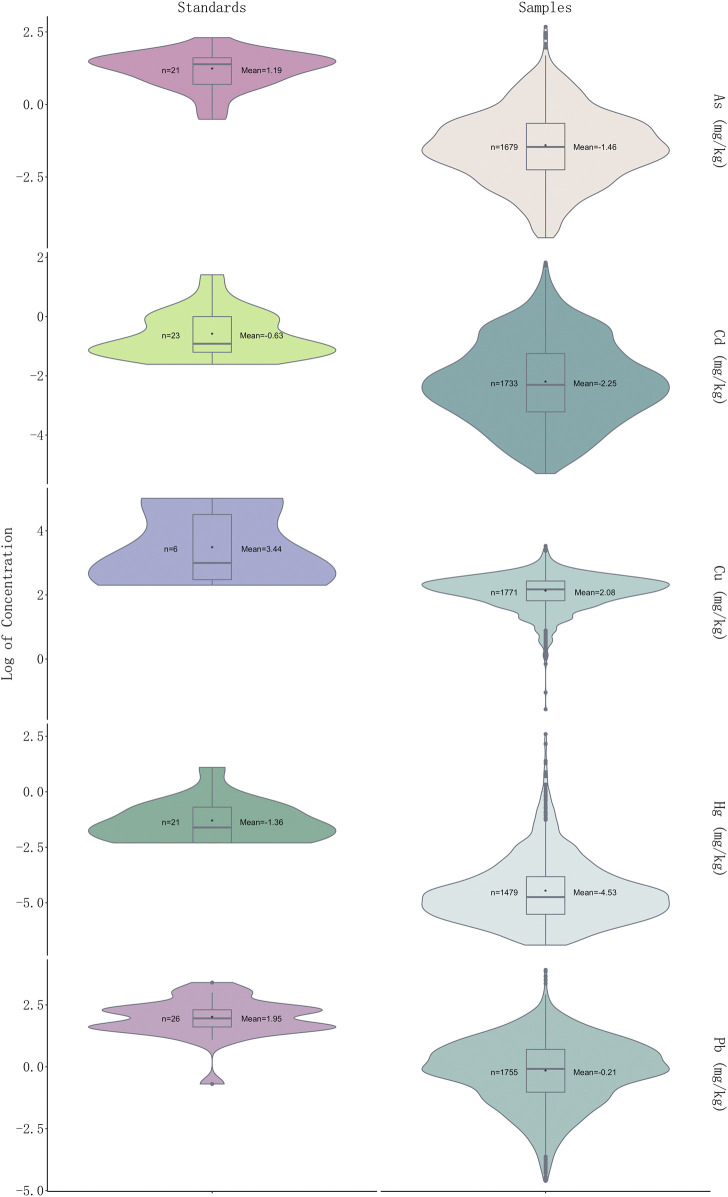
Violin plot showing log_2_ (concentration) values of five heavy metals in 1773 samples and permissible limits in different countries of each heavy metal (see Supplementary Table S3 for detailed standards of each heavy metal).

In a total of 541 samples (30.51%) were detected at levels over the CP (2020 edition) standard, and 433 samples detected with one over-limit metal, 75 samples with two over-limit metals, three samples with 24 over-limit metals and nine samples with four over-limit metals (Figure 2). The order of over-limit ratio of five heavy metals based on CP (2020 edition) standard is Pb (102, 5.75%) > Cd (88, 4.96%) > As (74, 4.17%)> Hg (67, 3.78%) > Cu (31, 1.75%). The times of highest concentration detected over the standards of CP (2020 edition) were 1.70 for Cu (*Schisandra chinensis* (Turcz.) Baill.), 6.27 for As (*Plantago asiatica* L.), 5.20 for Cd (*Curcuma longa* L.), 66.17 for Hg (*Chrysanthemum indicum* L.), and 9.02 for Pb (*Tetradium ruticarpum* (A.Juss.) T.G.Hartley). The highest concentration detected over the limit of European Union (EU) and United Kingdom (U.K.) is Hg (133.35 times). As per over-limit ratios of each metal in five medicinal properties, 40.00% samples were detected with over-limit concentrations in flos, 34.39% in folium and cortex, 7.69% in fructus and semen, 58.16% in herba and others, 37.20% in radix and rhizome. For Cu (*n* = 31), there are 12.90% samples detected over-limit in flos, 9.68% in folium and cortex, 16.13% in fructus and semen, 41.94% in herba and others, and 19.35% in radix and rhizome; For As (*n* = 74), 9.46% samples were detected with concentrations above the threshold in flos, 6.76% in folium and cortex, 8.11% in fructus and semen, 75.68% in herba and others, and none in radix and rhizome; For Cd (*n* = 416), 27.16% samples detected over-limit in flos, 10.10% in folium and cortex, 5.29% in fructus and semen, 39.90% in herba and others, and 17.55% in radix and rhizome; For Hg (*n* = 67), there are 25.37% samples detected over-limit in flos, 5.97% in folium and cortex, 34.33% in fructus and semen, 34.33% in herba and others, and none in radix and rhizome; For Pb (*n* = 102), there are 11.76% samples detected over-limit in flos, 9.80% in folium and cortex, 9.80% in fructus and semen, 59.80% in herba and others, and 8.82% in radix and rhizome. Of note, we found that heavy metals prefer to accumulate in fructus and semen, while herba and others were detected with the highest over-limit ratio. Notably, heavy metal Pb was presented with the highest over-limit ratio, followed by Cd and As.

### Risk Assessments of Five Heavy Metals in Herbal Medicines

**FIGURE 3:**
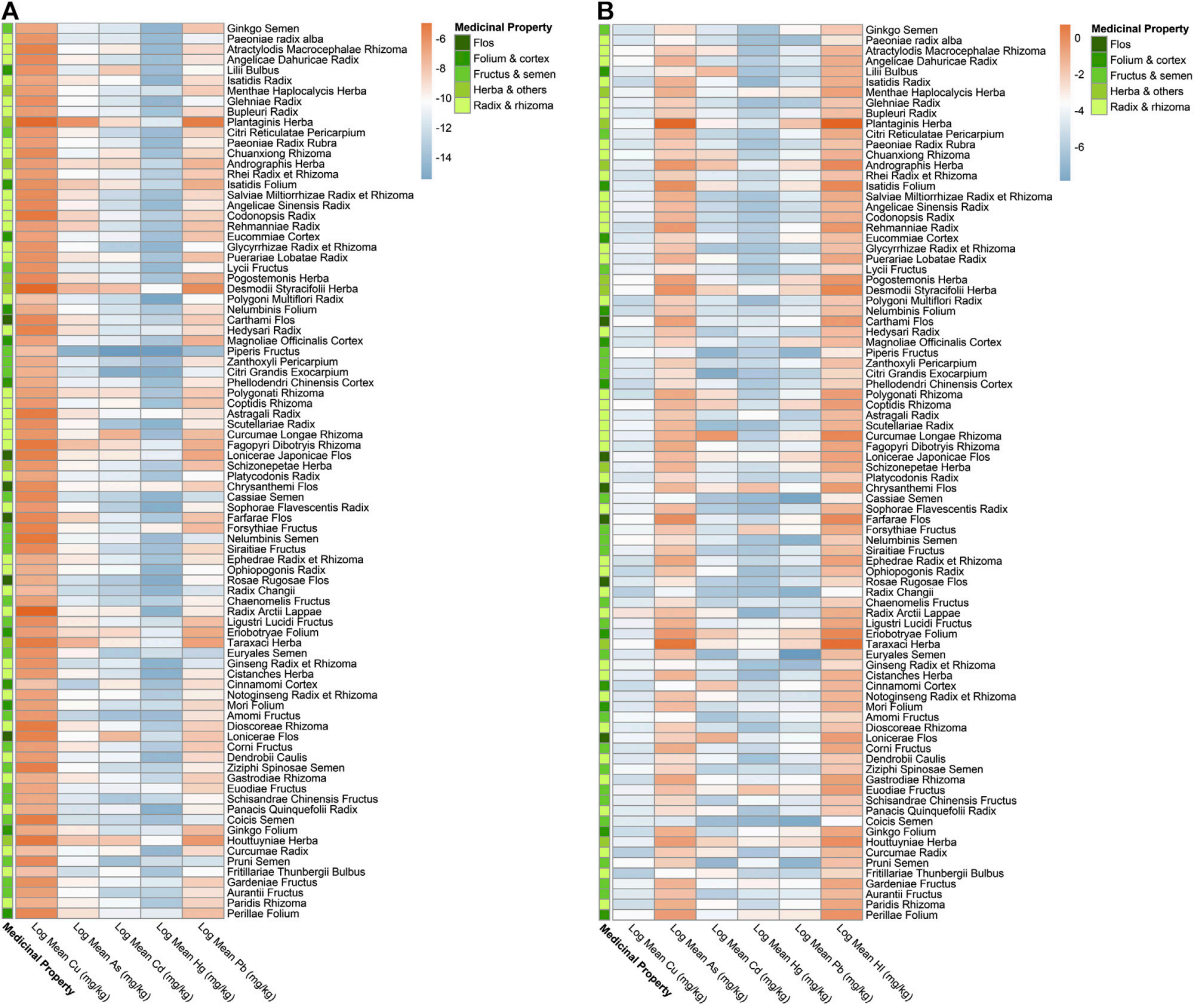
Heatmap of EDI and HI by mean concentrations detected for five heavy metals in 86 kinds of herbal medicines. **(A)** Heatmap showing log_2_ (Estimated Daily Intake, EDI) values by mean concentrations detected of five heavy metals in 86 herbal medicines. **(B)** Heatmap showing log_2_ (Hazard Index, HI) values by mean concentrations detected of five heavy metals in 86 herbal medicines.

As per exposure assessment, though the majority of herbal medicines were considered within the acceptable limit, the concentrations detected in all five heavy metals in few herbal medicines have surpassed their corresponding Provisional Tolerable Daily Intakes (PTDI), demonstrating unacceptable risk to health. The EDIs of three heavy metals (Cd, Hg, and Pb) in *Desmodii styracifolii* herba have surpassed their corresponding PTDIs (Figure 3; Supplementary Figure S1; Table 2). Notably, a total of 12 herbal medicines out of 86 have presented with EDIs above their corresponding PTDIs. The maximal EDI of As in three herbal plants *Plantago asiatica* L. (0.007), *Taraxacum officinale* (L.) Weber ex F.H.Wigg. (0.002), and *Cornus officinalis* Siebold and Zucc. (0.002); of Cd in five herbal plants *Desmodium styracifolium* (Osbeck) Merr. (0.001), *Andrographis paniculata* (Burm.f.) Nees (0.0008), *Curcuma longa* L. (0.001), *Lonicera japonica* Thunb. (0.001), and *Houttuynia cordata* Thunb. (0.002), of Hg in four herbal plants *Desmodium styracifolium* (Osbeck) Merr. (0.001), *Chrysanthemum indicum* L. (0.001), *Forsythia suspensa* (Thunb.)Vahl (0.001), and *Tetradium ruticarpum* (A.Juss.) T.G.Hartley (0.001); and of Pb in seven herbal plants *Plantago asiatica* L. (0.024), *Taraxacum officinale* (L.) Weber ex F.H.Wigg. (0.004), *Desmodium styracifolium* (Osbeck) Merr. (0.008) *Lonicera japonica* Thunb. (0.004), *Houttuynia cordata* Thunb. (0.004), *Tetradium ruticarpum* (A.Juss.) T.G.Hartley (0.004), *Lonicera japonica* Thunb. (0.005) exceeded their corresponding PTDIs (Figure 3).

**TABLE 2:** Health risk assessment scores of top risk-inducing herbal medicines.

Herbal medicine	Medicinal plant property	Max EDI (mg·kg^−1^·day^−1^·bw)				Max HQ				HI	Max CR			
		As	Cd	Hg	Pb	As	Cd	Hg	Pb		As	Cd	Pb	Total CR
*Mentha canadensis* L.	Herba and others					1.54				2.28				
*Plantago asiatica *L.	Herba and others	0.007			0.024	9.95			1.11	11.47	1.34E−06			
*Andrographis paniculata* (Burm.f.) Nees	Herba and others		0.001			5.51				7.02				2.18E−06
*Isatis tinctoria* L.	Folium and cortex					1.64				2.21				
*Pueraria montana* (Lour.) Merr.	Radix and rhizoma					1.03				1.35				
*Grona styracifolia* (Osbeck) H.Ohashi and K.Ohashi	Herba and others		0.001	0.001	0.008	1.79				3.47				
*Carthamus tinctorius* L.	Flos					2.02				2.41				
*Chrysanthemum indicum* L.	Flos			0.001		1.90		4.08		6.31				
*Tussilago farfara* L.	Flos					2.24		1.89		2.65				
*Forsythia suspensa* (Thunb.) Vahl	Fructus and semen			0.001		1.29				3.51				
*Ligustrum lucidum* W.T.Aiton	Fructus and semen					1.74				2.91				
*Taraxacum officinale* (L.) Weber ex F.H.Wigg.	Herba and others	0.002			0.004	6.82		`		8.62				
*Cornus officinalis* Siebold and Zucc.	Fructus and semen	0.002				8.05				8.66				
*Ziziphus jujuba* Mill.	Fructus and semen					1.58				1.83				
*Tetradium ruticarpum* (A.Juss.) T.G.Hartley	Fructus and semen			0.001	0.004	1.69		6.31	1.18	9.53			1.23E−07	
*Schisandra chinensis* (Turcz.) Baill.	Fructus and semen					1.07				1.49				
*Houttuynia cordata* Thunb.	Herba and others		0.002		0.004	1.97				3.42				
*Gardenia jasminoides* J.Ellis	Fructus and semen					2.93		1.14		4.57				
*Citrus × aurantium* L.	Fructus and semen					1.08				1.28				
*Perilla frutescens* (L.) Britton	Folium and cortex					2.64		1.02		4.02				
*Curcuma longa* L.	Radix and rhizoma		0.001				1.02			1.43		1.55E−06		
*Coptis chinensis* Franch.	Radix and rhizoma													
*Lonicera japonica* Thunb.	Flos				0.005					2.12				
*Chaenomeles lagenaria* (Loisel.) Koidz.	Fructus and semen									1.48				
*Lonicera confusa* DC.	Flos		0.001		0.004					1.81				

The estimated daily intakes (EDI, mg·kg^-1^·day^-1^·bw) above their corresponding provisional tolerable daily intakes (PTDI) were shown (The PTDIs (mg·kg^-1^·day^-1^) of As, Hg, Pb, and Cd are 0.00214, 0.00057, 0.00357, and 0.00083, respectively). The non-carcinogenic hazard quotient (HQ) and non-carcinogenic Hazard Index (HI) above one were shown. The carcinogenic risks (CR) higher than 10^-6^, which means one case of cancer over one million exposed people, is considered unacceptable, thus shown here. All scores in this table were calculated with maximal concentrations of each herbal medicine.

For non-carcinogenic risk, the majority of the herbal medicines were calculated with risks within the acceptable limit (<1). The HIs of a total of 86 herbal medicines ranged from 11.47 *Plantago asiatica* L. and 0.02 *Chaenomeles lagenaria* (Loisel.) Koidz. and HIs in a total of 25 out of 86 kinds of herbal medicines (29.07%) showed values over 1, thus considered unacceptable risk. HQs of As in 20 herbal medicines, of Hg in five herbal medicines, of Pb in two herbal medicines, and of Cd in one herbal medicine exceeded 1, considering as unacceptable risks. It was also shown that heavy metal As contributed the most in HQ > 1 herbal medicines. The highest was 92.94% in *Cornus officinalis* Siebold and Zucc. Heavy metal As has shown the highest non-carcinogenic (HQ = 9.95), presenting more severe risks than other four heavy metals (Figure 3; Table 2; Supplementary Figure S2).

**FIGURE 4:**
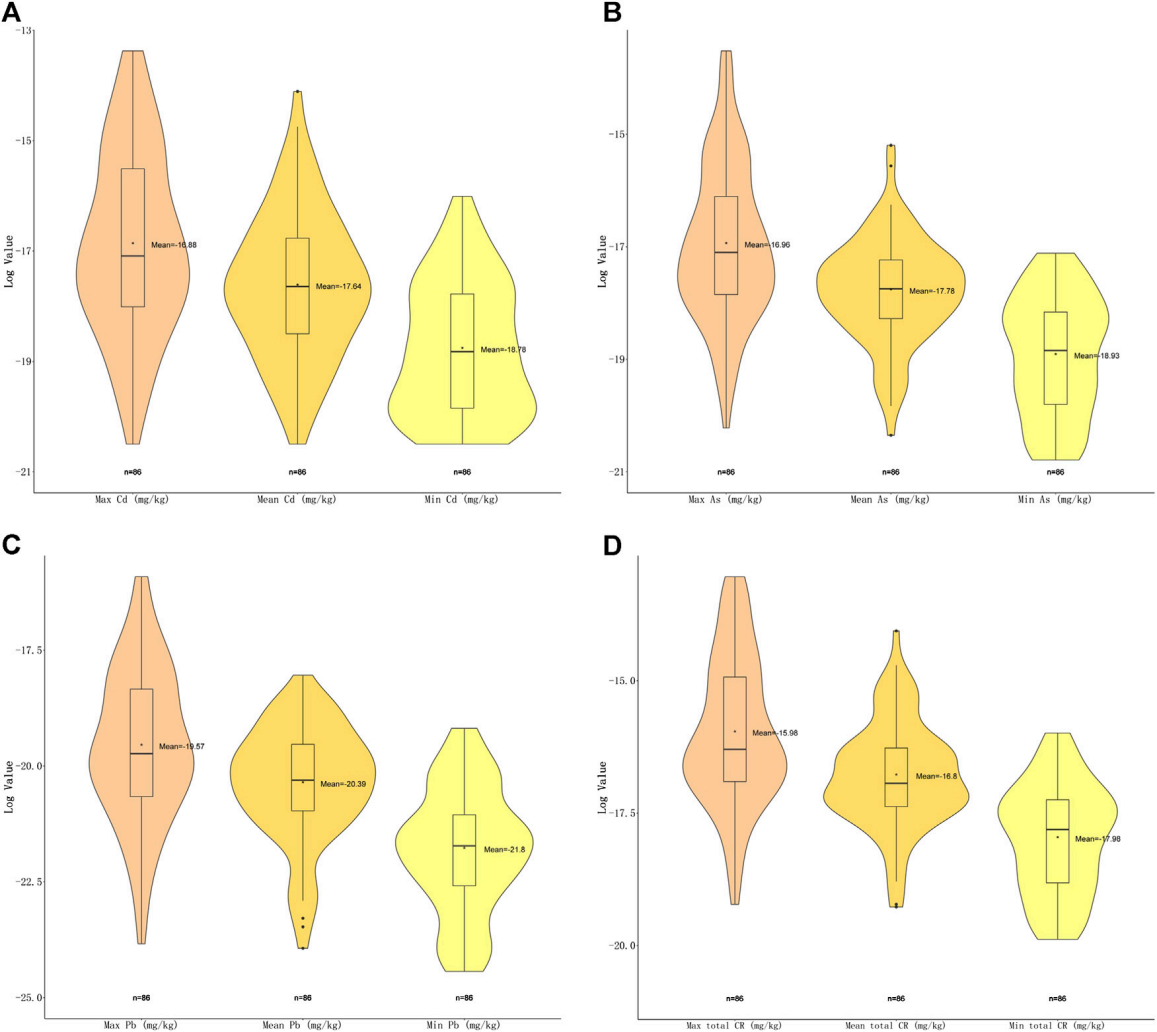
Violin plot of carcinogenic risk (CR) of three carcinogenic metals with maximal, minimal and mean concentrations detected. **(A)** Violin plot showing log_2_ (CR) values of Cd with maximal, minimal and mean concentrations. **(B)** Violin plot showing log_2_ (CR) values of As with maximal, minimal and mean concentrations. **(C)** Violin plot showing log_2_ (CR) values of Pb with maximal, minimal and mean concentrations **(D)** Violin plot showing total log_2_ (CR) values with maximal, minimal and mean concentrations.

For carcinogenic risks, all CRs were found to be within the acceptable limit (10^−4^) (Alidadi et al., 2019). The highest risk of As was found in *Plantago asiatica* L (1.34E−06), the lowest was in *Ziziphus jujuba* Mill. (9.36E−10); the highest of Cd was in *Curcuma longa* L. (1.55E−06), the lowest in *Citri Grandis Exocarpium* (1.25E−09); and the highest of Pb was in *Tetradium ruticarpum* (A.Juss.) T.G.Hartley (1.23E−07), the lowest were in *Pueraria montana* (Lour.) Merr. and *Ziziphus jujuba* Mill. (2.45E−11). *Andrographis paniculata* (Burm.f.) Nees presented with the highest total carcinogenic risk (2.18E−06), while *Ziziphus jujuba* Mill. the lowest (2.31E−09). Among these top risk-inducing herbs, nine belong to fructus and semen (Figure 4). Heavy metal As and Cd have shown more serious carcinogenic risks (CR = 1.34E−06, 1.55E−06).

In total here, 25 (29.07%) different kinds of herbal medicines (*n* = 86), presented with unacceptable risks based on exposure assessment, among which, nine belonged to fructus and semen, six belonged to herba and others, five belonged to the flos category, three belonged to radix and rhizoma, and two belonged to the folium and cortex. *Plantago asiatica* L. presented with the highest non-carcinogenic risk (HI = 11.47), while *Andrographis paniculata* (Burm.f.) Nees with the highest carcinogenic risk (CR = 2.18E−06). Heavy metal As has shown the highest non-carcinogenic (HQ = 9.95) while Cd the carcinogenic risk (1.55E−06) in herbal medicines. As these particular herbal medicines and heavy metals have the potential to cause health problems, they are in need of special monitoring to reduce potential risk (Table 2).

## Discussion

Based on this study with a large-spatio-temporal-scale herbal medicine samples, 30.51% (541) of samples were detected with at least one over-limit heavy metal. Five heavy metals (As, Pb, Cu, Hg and Cd) were widely detected in cultivated herbal medicines according to our experiment, which is in accordance with the other published results (Ernst, 2002; Kim et al., 2013). In Nigerian herbal remedies, 100% of the samples also contained elevated amounts of heavy metals (Obi et al., 2006), which revealed that the Nigerian herbs contained high levels of Fe, Ni, Cd, Cu, Pb, Se, and Zn sufficient to cause adverse health effect when regularly taken as recommended. In our study, 27 (31.40%) different kinds of herbal medicines, mostly with fructus and semen part with medicinal applications posed unacceptable health risk due to heavy metal accumulation though herba and others were detected with the highest over-limit ratio. Toxic element As posed the most serious health risk according to exposure, carcinogenic and non-carcinogenic risk assessments, as health risk assessment employed by Ren, indicated that As and Pb generated from industrial sites and traffic sites has a potential to pose serious health risks (Saifullah et al., 2015). It was also found that As was the major metal found for water pollution (Alidadi et al., 2019), exceeding its permitted daily exposure dosages and suggested a potential health risk for *Panax notoginseng* (Burkill) F.H.Chen consumers (Eisenberg et al., 2018). Therefore, we can conclude that As might be the one of five heavy metals necessitating special attention, possibly due to various states of As accumulation and absorption from soil and water. Furthermore, Bolan employed a study and asserted that the concentrations of Cd, Hg, and Pb in Ayurvedic medicines exceeded their daily intake amounts (Bolan et al., 2017). According to the study by Lee SD, levels of Cd exceeding WHO reference values were observed in 10 samples and the weekly intakes of Pb, Cd, Cr, Cu, Hg from herbs (Lee et al., 2012). While in Iran, maximum bioaccumulate of Pb and Hg was noted in *artemisia dracunculus* L. and *Spinacia oleracea* L., respectively (Fu et al., 2018). The non-carcinogenic risks target hazard quotients (THQs) of Al and Cr from individual herbs were over 1, which might impart risk for human consumption (Kohzadi et al., 2018). It could be concluded that heavy metals As, Pb, Cd, and Hg all impose significant risk to health due to herbal consumption. The highest HI was presented in herbal medicine *Plantago asiatica* L. (HI = 11.47) and highest CR in *Andrographis paniculata* (Burm.f.) Nees (CR = 2.18E−06), which are in need of special dosage control and monitoring. Furthermore, the highest over-limit ratios of five heavy metals based on different producing areas are Cu (7.69%) in Chongqing, As (20.21%) in Gansu province, Cd (0.77%) in Chongqing, Hg (9.89%) in Hunan province, and Pb (25.00%) in Fujian province (Supplementary Table S3). However, Principle Components Analysis (PCA) didn’t showcase that there was significant statistic difference regarding the five heavy mental accumulations in five medicinal plant properties. While according to Pearson Correlation Analysis, Pb and As were correlated to flos, folium and cortex, fructus and semen, and herba and others, while Pb and Cd in radix and rhizoma (Supplementary Figures S1, S2). Additionally, based on the Analysis of Similarities (ANOSIM), it was indicated that the difference within groups of five medicinal plant properties is less significant than the one throughout the five groups (R = 0.165, *p* = 0.001) (Supplementary Figures S3, S5).

Heavy metals may induce a variety of pathologies damaging our health. They may also react directly with DNA, inducing a variety of DNA lesions including both DNA strand damage and DNA protein cross-linkage (Harris et al., 2011). Generally, five reasons explain the levels of heavy metal contents in herbal medicines. The first is the variable exposure to environmental pollution including industrial encroachment, contaminated soil or atmosphere (Filipiak-Szok et al., 2015). The physicochemical properties of soil including pH, temperature, redox potential, translocation exchange capacity and organic matter may influence the availability of metal to plants. Secondly, the phytological characteristics of medical plants themselves such as reduced biomass, root length and shoot length are common indicators of heavy metal toxicity. Furthermore, the interactions of soil-plant roots-microbes play vital roles in regulating heavy metal movement from the soil to edible plant parts. Certain plants are “hyper-accumulators” which grow on metalliferous soils and accumulate extraordinarily high levels of heavy metals without displaying phytotoxic effects. Thirdly, herbal plants could be contaminated during manufacturing and agronomic processes (Oliveira et al., 2018) including growing, harvesting, transportation, processing and storage, due to pesticide formulations, chemical fertilizers and irrigation with poor-quality water. (Harris et al., 2011; Bolan et al., 2017; Filipiak-Szok et al., 2015). For example, Cd and Pb may enter the soil due to fertilizer impurities (Rai, 2012), non-ferrous smelters, lead and zinc mines, sewage slug application and combustion of fossil fuels (Khan et al., 2008). Additionally, fumigants containing heavy metals may also be applied for preventing rats and mildew (Fujita et al., 2016). Fourthly, plant uptake is one of the major routes of dietary exposure to heavy metals in the soil, and the wide variations in metal concentrations in the analyzed herbs could be attributed to differences in the plant metal uptake and translocation capabilities. Studies have shown wide variations in concentration factor for different metals among different plant species and sampling sites. Certain species have higher tendency to accumulate Cd (Harris et al., 2011). Lastly, the bioavailability of heavy metals could have an impact on their concentrations, such as soil pH, the metal levels already resident in the soil, the oxidation-reduction potential of the soil, and other chemical and physical factors (Supplementary Table S4).

**FIGURE 5:**
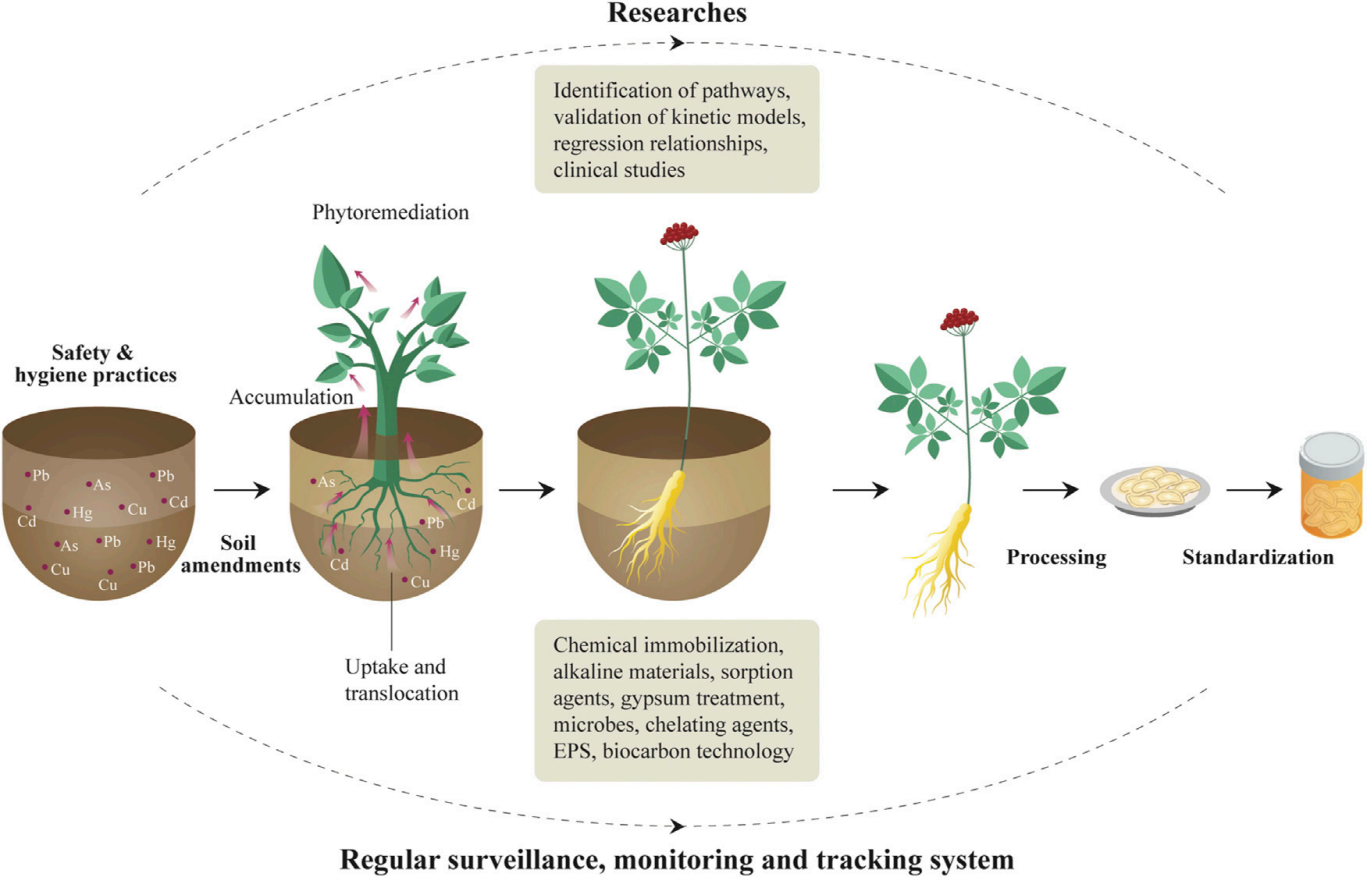
From-cradle-to-grave heavy metal control strategy of herbal medicine production.

Here we propose a solution for heavy metal control in herbal medicines (Figure 5). We consider, given the results found here and those of others previously there is an urgent need to implement a regular monitoring and surveillance program, controlling extrinsic contamination of herbal medicines along the supply chain from field to consumer (Bolan et al., 2017). Secondly, research, such as identifying ways in which heavy metals reach herbal products; development and validation of kinetic models linking processing techniques with metal speciation and bioavailability; bioavailability tests of heavy metals in herbal medicines; experiments on regression relationships between speciation and bioavailability of heavy metals, clinical studies examining the toxicity of heavy metals, etc. (Harris et al., 2011). Soil amendments, including mitigation and preservation management for the growth performance of biomass and metal accumulation in contaminated soils, is necessary (Kim et al., 2016). Thirdly, international and universal standards related to risk assessments and further permissible limits are in urgent need. For example, the transfer rate of heavy metals varies greatly among medicinal materials, but there is no international guideline that gives a general rule. While in our research, we calculated all the risks with minimal, mean, and maximal concentrations of five heavy metals detected in each kind of herbal medicines to cover the most-likely scenarios to the worst-case scenarios. Therefore, a universal transfer rate is necessarily needed and will be convenient for the development of international standards. Lastly, tolerant medicinal plants with high phytoremediation potential (Khattak et al., 2015) and capability for phytostabilization and phytoextraction (Kim et al., 2016) can be cultivated as an approach for the management and targeted bio-extraction of heavy metals from moderately polluted lands (Fakhari et al., 2017), together with a combination of different agents such as pH change-inducing chemical immobilization, alkaline materials including lime based materials, fly ash, and biochar, calcite, dolomite, oyster and egg shell (Kim et al., 2016). Sorption agents such as phosphate materials, compost, zeolite and iron compounds, activated carbon, and bentonite, or materials that decrease dissolved organic carbon such as gypsum treatment, Solanum nigrum, microbes, chelating agents, Extracellular polysaccharides or Exopolysaccharides (Rehman et al., 2017), and eco-friendly biocarbon technology (Augustina and Adriana, 2014). These materials increase soil pH, favor deprotonation and the formation of oxides, metal-carbonate precipitates, complexes and secondary minerals that all decrease the phytoavailable heavy metal concentrations (Maiga et al., 2005; Kranthi et al., 2018). Phytoremediation has been perceived to be a more low-cost, low-impact, low-tech alternative, visually benign and environmentally sound comparing to more active and intrusive remedial methods (Augustina and Adriana, 2014).

In conclusion, heavy metal contamination in herbal medicines was borderline or higher than the safety level. There are 30.51% samples detected with at least one over-limit heavy metal according to Chinese Pharmacopoeia (CP, 2020 edition) standards (National Pharmacopoeia Commission, 2020). The risk assessments have demonstrated that the majority (70.93%) of the herbal plants were within acceptable risks. Notably, As posed the highest risk in all indicators including EDI, HI, and CR, inducing the most serious risks in all five metals. Herbal medicines *Tetradium ruticarpum* (A.Juss.) T.G.Hartley, *Plantago asiatica* L., and *Desmodium styracifolium* (Osbeck) Merr. were considered the most risk-inducing herbal medicines. Contamination in herbal medicines is well demonstrated and clearly poses a serious potential risk to health. Furthermore, trace metals play a significant role in reactions which lead to formation of the active chemical plant constituents and are, therefore, responsible in-part for their curative as well as toxic properties. The analysis of toxic metals can be useful to evaluate the dosage of the herbal drugs prepared from these plants (Parveen et al., 2013). Therefore, it is of great advantage to establish universal standards and quality requirements for hazardous elements in herbal medicines so that this natural resource can continue and expand further, to benefit health globally (Luo et al., 2019).

## Data Availability Statement

The raw data supporting the conclusions of this article will be made available by the authors, without undue reservation.

## Author Contributions

SC and LD conceived the study. LD and LL collected and sorted the samples. BW conducted the heavy metal analysis. LL, JJ, MF, QH, and ZY analyzed the data. LL, JJ, QH, HL, and JZ plotted the figures. LL, JW, CY, and HZ created the tables and supporting materials. SC, LD, and LL contributed to drafting the first version of the manuscript, and all authors proved the final text.

## Funding

This projected was supported by Beijing Nova Program (Z181100006218020), Fundamental Research Funds for the central public welfare research institutes (No. ZZ13-AQ-049), Major science and technology projects of Yunnan province (2018ZF011), National Key R&D Plan (2018YFC1706302), National Science and Technology Major Project for “Significant New Drugs Development” (2019ZX09201005-006-001), and National Natural Science Foundation (No. 81973429).

## Conflict of Interest

The authors declare that the research was conducted in the absence of any commercial or financial relationships that could be construed as a potential conflict of interest.

The reviewer JC declared a past co-authorship with one of the authors SC to the handling editor.
