# Supplementary material for: Heavy Metal Contaminations in Herbal Medicines: Determination, Comprehensive Risk Assessments, and Solutions
Source: Front Pharmacol. 2021 Jan 14;11:595335. doi: 10.3389/fphar.2020.595335 (PMC7883644; doi:10.3389/fphar.2020.595335)
Supplement: Supplementary file 1 [file datasheet1.docx]

**Appendix**

**Heavy Metal Contaminations in Herbal Medicines: Determination, Comprehensive Risk Assessments, and Solutions**

This document provides supplementary materials for the manuscript Heavy Metal Contaminations in Herbal Medicines: Determination, Comprehensive Risk Assessments, and Solutions. The content of this document is intended to provide additional data and information to complement the content of the manuscript.

### Table of contents

Table of contents 1

Table S1.1 Methodological verification of ICP-MS for determination of five heavy metals in *Mentha canadensis* L. (18#) herbal medicines (n=3) 2

Table S1.2 Methodological verification of ICP-MS for determination of five heavy metals in Menthae *Andrographis paniculata* (Burm.f.) Nees (12#) herbal medicines (n=3) 3

Table S1.3 Methodological verification of ICP-MS for determination of five heavy metals in *Isatis tinctoria* L. (14#) herbal medicines (n=3) 3

Table S1.4 Methodological verification of ICP-MS for determination of five heavy metals in *Lycium barbarum* L. (10#) herbal medicines (n=3) 3

Table S1.5 Methodological verification of ICP-MS for determination of five heavy metals in *Grona styracifolia* (Osbeck) H.Ohashi & K.Ohashi (9#) herbal medicines (n=3) 4

Table S1.6 Methodological verification of ICP-MS for determination of five heavy metals in *Carthamus tinctorius* L. (4#) herbal medicines (n=3) 4

Table S1.7 Methodological verification of ICP-MS for determination of five heavy metals in *Lonicera japonica* Thunb. (19#) herbal medicines (n=3) 4

Table S1.8 Methodological verification of ICP-MS for determination of five heavy metals in *Chrysanthemum indicum* L. (26#) herbal medicines (n=3) 5

Table S1.9 Methodological verification of ICP-MS for determination of five heavy metals in *Tussilago farfara* L. (7#) herbal medicines (n=3) 5

Table S1.10 Methodological verification of ICP-MS for determination of five heavy metals in *Forsythia suspensa* (Thunb.) Vahl (6#) herbal medicines (n=3) 5

Table S1.11 Methodological verification of ICP-MS for determination of five heavy metals in *Chaenomeles lagenaria* (Loisel.) Koidz. (3#) herbal medicines (n=3) 6

Table S1.12 Methodological verification of ICP-MS for determination of five heavy metals in *Ligustrum lucidum* W.T.Aiton (2#) herbal medicines (n=3) 6

Table S1.13 Methodological verification of ICP-MS for determination of five heavy metals in *Taraxacum officinale* (L.) Weber ex F.H.Wigg. (27#) herbal medicines (n=3) 6

Table S1.14 Methodological verification of ICP-MS for determination of five heavy metals in *Lonicera confusa* DC. (13#) herbal medicines (n=3) 7

Table S1.15 Methodological verification of ICP-MS for determination of five heavy metals in *Cornus officinalis* Siebold & Zucc. (16#) herbal medicines (n=3) 7

Table S1.16 Methodological verification of ICP-MS for determination of five heavy metals in *Ziziphus jujuba* Mill. (1#) herbal medicines (n=3) 7

Table S1.17 Methodological verification of ICP-MS for determination of five heavy metals in *Tetradium ruticarpum* (A.Juss.) T.G.Hartley (8#) herbal medicines (n=3) 8

Table S1.18 Methodological verification of ICP-MS for determination of five heavy metals in *Schisandra chinensis* (Turcz.) Baill. (10#) herbal medicines (n=3) 8

Table S1.19 Methodological verification of ICP-MS for determination of five heavy metals in *Houttuynia cordata* Thunb. (2#) herbal medicines (n=3) 8

Table S1.20 Methodological verification of ICP-MS for determination of five heavy metals in *Gardenia jasminoides* J.Ellis (14#) herbal medicines (n=3) 9

Table S1.21 Methodological verification of ICP-MS for determination of five heavy metals in *Citrus × aurantium* L. (4#) herbal medicines (n=3) 9

Table S1.22 Methodological verification of ICP-MS for determination of five heavy metals in *Perilla frutescens* (L.) Britton (10#) herbal medicines (n=3) 9

Table S1.23 Methodological verification of ICP-MS for determination of five heavy metals in *Plantago asiatica* L. (4#) herbal medicines (n=3) 10

Table S2 Permissible limits of heavy metals in medicinal herbs (products) from different standards 10

Table S3 Over-limit ratio (%) of five heavy metals in 32 producing areas 12

Table S4 Chronic and acute adverse effects of five heavy metals on health 13

Fig.S1. The over-limit Estimated Daily Intakes (EDI) of four heavy metals in herbal medicines. 17

Fig.S2. The over-limit Hazard Quotients (HQ) of four heavy metals in 21 herbal medicines. 18

Fig.S3.1 Spearman correlation coefficient of metal relations in flos 19

Fig.S3.2 Spearman correlation coefficient of metal relations in folium & cortex 20

Fig.S3.3 Spearman correlation coefficient of metal relations in fructus & semen 21

Fig.S3.4 Spearman correlation coefficient of metal relations in herba & others 22

Fig.S3.5 Spearman correlation coefficient of metal relations in radix & rhizoma 23

Fig.S4.1 Principle Components Analysis (PCA) of five heavy metal contents in five plant properties 24

Fig.S4.2 PCA of five heavy metals in 32 producing areas 24

Fig.S5 Analysis of Similarities (ANOSIM) of five heavy metal contents in five medicinal plant properties 25

### Table S1.1 Methodological verification of ICP-MS for determination of five heavy metals in *Mentha canadensis* L. (18#) herbal medicines (n=3)

| **Heavy metals** | **Calibration curve** | ***r*** | **Repeatability RSD** | **Spike-and-recovery** | **Recovery RSD** | **LOD (µg·L^-1)^** |
| --- | --- | --- | --- | --- | --- | --- |
| **Pb** | *Y*=11148.3*X* +8097.5 | 0.9995 | 3.9% | 98.6% | 11.9% | 3×10^-6^ |
| **Cd** | *Y* =760.5*X*+18.0 | 0.9998 | 3.2% | 105.1% | 10.5% | 5×10^-5^ |
| **As** | *Y*=315.8*X* +510.0 | 0.9997 | 1.3% | 103.3% | 5.9% | 1×10^-3^ |
| **Hg** | *Y* =1700.8*X* +55.0 | 0.9998 | 8.6% | 76.5% | 7.7% | 2×10^-5^ |
| **Cu** | *Y* =750.1*X* +490.5 | 0.9998 | 0.6% | 81.7% | 3.6% | 7×10^-4^ |

*RDS is short for relative standard deviation

### Table S1.2 Methodological verification of ICP-MS for determination of five heavy metals in *Andrographis paniculata* (Burm.f.) Nees (12#) herbal medicines (n=3)

| **Heavy metals** | **Calibration curve** | ***r*** | **Repeatability RSD** | **Spike-and-recovery** | **Recovery RSD** | **LOD (µg·L^-1)^** |
| --- | --- | --- | --- | --- | --- | --- |
| **Pb** | *Y*=11148.3*X* +8097.5 | 0.9995 | 1.7% | 95.4% | 6.4% | 3×10^-6^ |
| **Cd** | *Y* =760.5*X*+18.0 | 0.9998 | 2.5% | 93.4% | 9.7% | 5×10^-5^ |
| **As** | *Y*=315.8*X* +510.0 | 0.9997 | 6.3% | 102.9% | 8.2% | 1×10^-3^ |
| **Hg** | *Y* =1700.8*X* +55.0 | 0.9998 | 3.5% | 95.1% | 4.6% | 2×10^-5^ |
| **Cu** | *Y* =750.1*X* +490.5 | 0.9998 | 1.8% | 96.7% | 2.9% | 7×10^-4^ |

### Table S1.3 Methodological verification of ICP-MS for determination of five heavy metals in *Isatis tinctoria* L. Folium (14#) herbal medicines (n=3)

| **Heavy metals** | **Calibration curve** | ***r*** | **Repeatability RSD** | **Spike-and-recovery** | **Recovery RSD** | **LOD (µg·L^-1)^** |
| --- | --- | --- | --- | --- | --- | --- |
| **Pb** | *Y*=19504.7*X* +4581.6 | 1.0000 | 0.5% | 102.9% | 0.7% | 3×10^-6^ |
| **Cd** | *Y* =1386.5*X*+39.3 | 1.0000 | 1.3% | 105.6% | 6.8% | 2×10^-5^ |
| **As** | *Y*=585.6*X* +421.6 | 0.9996 | 4.9% | 108.4% | 1.0% | 9×10^-4^ |
| **Hg** | *Y* =2664.9*X* +84.0 | 0.9998 | 7.1% | 101.1% | 9.9% | 3×10^-5^ |
| **Cu** | *Y* =1291.1*X* +795.5 | 1.0000 | 0.3% | 86.3% | 0.8% | 1×10^-4^ |

### Table S1.4 Methodological verification of ICP-MS for determination of five heavy metals in *Lycium barbarum* L. (10#) herbal medicines (n=3)

| **Heavy metals** | **Calibration curve** | ***r*** | **Repeatability RSD** | **Spike-and-recovery** | **Recovery RSD** | **LOD (µg·L^-1)^** |
| --- | --- | --- | --- | --- | --- | --- |
| **Pb** | *Y*=12993.9*X* +5823.1 | 1.0000 | 7.2% | 80.1% | 2.2% | 6×10^-5^ |
| **Cd** | *Y* =908.3*X*+17.4 | 1.0000 | 1.4% | 104.3% | 2.5% | 3×10^-5^ |
| **As** | *Y*=384.2*X* +606.2 | 0.9998 | 7.8% | 103.1% | 1.3% | 8×10^-4^ |
| **Hg** | *Y* =2019.4*X* +207.4 | 0.9999 | 11.2% | 86.6% | 4.1% | 1×10^-5^ |
| **Cu** | *Y* =901.1*X* +463.0 | 1.0000 | 7.7% | 91.0% | 11.6% | 2×10^-4^ |

### Table S1.5 Methodological verification of ICP-MS for determination of five heavy metals in *Grona styracifolia* (Osbeck) H.Ohashi & K.Ohashi (9#) herbal medicines (n=3)

| **Heavy metals** | **Calibration curve** | ***r*** | **Repeatability RSD** | **Spike-and-recovery** | **Recovery RSD** | **LOD (µg·L^-1)^** |
| --- | --- | --- | --- | --- | --- | --- |
| **Pb** | *Y*=11015.2*X* +11215.6 | 1.0000 | 1.3% | 82.0% | 6.9% | 2×10^-6^ |
| **Cd** | *Y* =824.8*X*+13.0 | 0.9999 | 1.2% | 102.7% | 1.5% | 2×10^-5^ |
| **As** | *Y*=340.4*X* +682.7 | 0.9997 | 2.1% | 99.4% | 9.1% | 8×10^-4^ |
| **Hg** | *Y* =1595.0*X* +31.0 | 0.9989 | 10.9% | 101.7% | 4.4% | 5×10^-5^ |
| **Cu** | *Y* =795.2*X* +3572.4 | 0.9999 | 5.9% | 77.3% | 9.8% | 5×10^-4^ |

### Table S1.6 Methodological verification of ICP-MS for determination of five heavy metals in *Carthamus tinctorius* L. (4#) herbal medicines (n=3)

| **Heavy metals** | **Calibration curve** | ***r*** | **Repeatability RSD** | **Spike-and-recovery** | **Recovery RSD** | **LOD (µg·L^-1)^** |
| --- | --- | --- | --- | --- | --- | --- |
| **Pb** | *Y* =17177.7*X*+14214.5 | 0.9999 | 7.7% | 90.7% | 2.4% | 2×10^-6^ |
| **Cd** | *Y*= 1145.4*X* +28.0 | 1.0000 | 7.3% | 106.4% | 4.2% | 2×10^-5^ |
| **As** | *Y* =473.2*X* +1057.8 | 0.9994 | 9.8% | 113.7% | 5.3% | 5×10^-4^ |
| **Hg** | *Y* =2743.9*X* + 67.99 | 0.9999 | 5.6% | 92.4% | 5.7% | 10^-5^ |
| **Cu** | *Y*= 1091.4*X* + 1018 | 1.0000 | 0.5% | 88.6% | 2.6% | 2×10^-4^ |

### Table S1.7 Methodological verification of ICP-MS for determination of five heavy metals in *Lonicera japonica* Thunb. (19#) herbal medicines (n=3)

| **Heavy metals** | **Calibration curve** | ***r*** | **Repeatability RSD** | **Spike-and-recovery** | **Recovery RSD** | **LOD (µg·L^-1)^** |
| --- | --- | --- | --- | --- | --- | --- |
| **Pb** | *Y*=17177.7*X* +14214.5 | 0.9999 | 0.3% | 113.4% | 7.1% | 2×10^-6^ |
| **Cd** | *Y* =1145.4*X*+28.0 | 1.0000 | 0.8% | 109.4% | 2.4% | 2×10^-5^ |
| **As** | *Y*= 473.2*X* +1057.8 | 0.9994 | 3.2% | 109.1% | 1.1% | 5×10^-4^ |
| **Hg** | *Y* =2380.8*X* +1101.9 | 0.9997 | 8.6% | 88.1% | 8.3% | 3×10^-5^ |
| **Cu** | *Y* =1091.4*X* +1018.0 | 1.0000 | 0.6% | 88.5% | 6.9% | 2×10^-4^ |

### Table S1.8 Methodological verification of ICP-MS for determination of five heavy metals in *Chrysanthemum indicum* L. (26#) herbal medicines (n=3)

| **Heavy metals** | **Calibration curve** | ***r*** | **Repeatability RSD** | **Spike-and-recovery** | **Recovery RSD** | **LOD (µg·L^-1)^** |
| --- | --- | --- | --- | --- | --- | --- |
| **Pb** | *Y* =10197.19*X* +5586.1 | 0.9999 | 6.3% | 75.1% | 4.7% | 3×10^-6^ |
| **Cd** | *Y*= 972.26*X* +151.18 | 1.0000 | 10.1% | 101.5% | 3.4% | 6×10^-5^ |
| **As** | *Y* =398.76 *X* +1153.0 | 0.9991 | 7.2% | 104.4% | 3.7% | 6×10^-4^ |
| **Hg** | *Y* =1806.18*X* + 72.68 | 0.9999 | 8.1% | 93.6% | 8.7% | 1×10^-5^ |
| **Cu** | *Y*= 1001.31*X* +1208.08 | 1.0000 | 2.5% | 86.2% | 1.8% | 2×10^-4^ |

### Table S1.9 Methodological verification of ICP-MS for determination of five heavy metals in *Tussilago farfara* L. (7#) herbal medicines (n=3)

| **Heavy metals** | **Calibration curve** | ***r*** | **Repeatability RSD** | **Spike-and-recovery** | **Recovery RSD** | **LOD (µg·L^-1)^** |
| --- | --- | --- | --- | --- | --- | --- |
| **Pb** | *Y* =11509.6*X* +9428.9 | 0.9995 | 8.5% | 91.8% | 1.2% | 3×10^-6^ |
| **Cd** | *Y*= 748.6*X* +26 | 1.0000 | 3.5% | 98.8% | 4.0% | 5×10^-5^ |
| **As** | *Y* =317.9*X* +498.7 | 0.9996 | 6.5% | 93.0% | 8.5% | 1×10^-3^ |
| **Hg** | *Y* =1665.6*X* + 239 | 0.9989 | 6.4% | 89.4% | 6.8% | 2×10^-5^ |
| **Cu** | *Y*=760.2*X*+758.7 | 1.0000 | 2.8% | 79.9% | 8.5% | 7×10^-5^ |

### Table S1.10 Methodological verification of ICP-MS for determination of five heavy metals in *Forsythia suspensa* (Thunb.) Vahl (6#) herbal medicines (n=3)

| **Heavy metals** | **Calibration curve** | ***r*** | **Repeatability RSD** | **Spike-and-recovery** | **Recovery RSD** | **LOD (µg·L^-1)^** |
| --- | --- | --- | --- | --- | --- | --- |
| **Pb** | *Y*=11148.3*X* +8097.5 | 0.9995 | 1.3% | 109.4% | 6.0% | 3×10^-6^ |
| **Cd** | *Y* =760.5*X*+18.0 | 0.9998 | 4.7% | 104.1% | 3.9% | 5×10^-5^ |
| **As** | *Y*=315.8*X* +510.0 | 0.9997 | 11.8% | 100.1% | 2.7% | 1×10^-3^ |
| **Hg** | *Y* =1700.8*X* +55.0 | 0.9998 | 10.9% | 79.0% | 8.2% | 2×10^-5^ |
| **Cu** | *Y* =750.1*X* +490.5 | 0.9998 | 1.3% | 78.7% | 1.5% | 7×10^-4^ |

### Table S1.11 Methodological verification of ICP-MS for determination of five heavy metals in Chaenomelis Fructus (3#) herbal medicines (n=3)

| **Heavy metals** | **Calibration curve** | ***r*** | **Repeatability RSD** | **Spike-and-**  **recovery** | **Recovery RSD** | **LOD (µg·L^-1)^** |
| --- | --- | --- | --- | --- | --- | --- |
| **Pb** | *Y* =16117.4*X* +19882 | 0.9999 | 2.0% | 96.6% | 0.1% | 5×10^-5^ |
| **Cd** | *Y*= 1262.5*X* +23.96 | 1.0000 | 4.8% | 92.4% | 0.6% | 2×10^-5^ |
| **As** | *Y* =582.8 *X* +1129.9 | 0.9999 | 8.5% | 97.2% | 1.9% | 5×10^-4^ |
| **Hg** | *Y* =2743.9*X* + 67.99 | 0.9999 | 7.7% | 71.5% | 5.9% | 10^-5^ |
| **Cu** | *Y*= 1319.1*X* + 2240.4 | 0.9999 | 2.1% | 85.8% | 2.6% | 10^-4^ |

### Table S1.12 Methodological verification of ICP-MS for determination of five heavy metals in *Ligustrum lucidum* W.T.Aiton (2#) herbal medicines (n=3)

| **Heavy metals** | **Calibration curve** | ***r*** | **Repeatability RSD** | **Spike-and-**  **recovery** | **Recovery RSD** | **LOD (µg·L^-1)^** |
| --- | --- | --- | --- | --- | --- | --- |
| **Pb** | *Y* =20110.3*X* +4383.6 | 1.00008 | 5.1% | 83.1% | 5.8% | 6×10^-7^ |
| **Cd** | *Y*= 1527.02*X* +64.0 | 1.0000 | 2.5% | 108.9% | 1.8% | 2×10^-5^ |
| **As** | *Y* =668.34 *X* +1189.4 | 0.9996 | 4.0% | 114.3% | 2.3% | 3×10^-4^ |
| **Hg** | *Y* =2884.93*X* + 66.63 | 0.9999 | 11.2% | 92.7% | 7.5% | 10^-5^ |
| **Cu** | *Y*= 1554.11*X* + 1172.2 | 1.0000 | 1.7% | 88.5% | 2.6% | 2×10^-4^ |

### Table S1.13 Methodological verification of ICP-MS for determination of five heavy metals in *Taraxacum officinale* (L.) Weber ex F.H.Wigg. (27#) herbal medicines (n=3)

| **Heavy metals** | **Calibration curve** | ***r*** | **Repeatability RSD** | **Spike-and-recovery** | **Recovery RSD** | **LOD (µg·L^-1)^** |
| --- | --- | --- | --- | --- | --- | --- |
| **Pb** | *Y*=19085.2*X* +4821.7 | 0.9998 | 1.4% | 102.2% | 1.7% | 8×10^-7^ |
| **Cd** | *Y* =1301.8*X*+45.5 | 1.0000 | 3.8% | 105.8% | 2.3% | 2×10^-5^ |
| **As** | *Y*=531.1*X* +423.4 | 0.9997 | 5.6% | 97.7% | 6.8% | 1×10^-3^ |
| **Hg** | *Y* =2604.7*X* +295.2 | 1.0000 | 8.5% | 79.6% | 8.3% | 1×10^-5^ |
| **Cu** | *Y* =1166.0*X* +913.9 | 1.0000 | 1.4% | 91.2% | 2.9% | 1×10^-4^ |

### Table S1.14 Methodological verification of ICP-MS for determination of five heavy metals in *Lonicera confusa* DC. (13#) herbal medicines (n=3)

| **Heavy metals** | **Calibration curve** | ***r*** | **Repeatability RSD** | **Spike-and-recovery** | **Recovery RSD** | **LOD (µg·L^-1)^** |
| --- | --- | --- | --- | --- | --- | --- |
| **Pb** | Y=17177.7*X* +14214.5 | 0.9999 | 3.1% | 104.4% | 3.9% | 2×10^-6^ |
| **Cd** | Y =1145.4*X*+28.0 | 1.0000 | 2.0% | 106.4% | 6.3% | 2×10^-5^ |
| **As** | Y= 473.2*X* +1057.8 | 0.9994 | 0.7% | 108.1% | 5.3% | 5×10^-4^ |
| **Hg** | Y =2380.8*X* +1101.9 | 0.9997 | 10.0% | 89.1% | 7.3% | 3×10^-5^ |
| **Cu** | Y =1091.4*X* +1018.0 | 1.0000 | 2.3% | 87.9% | 9.9% | 2×10^-4^ |

### Table S1.15 Methodological verification of ICP-MS for determination of five heavy metals in *Cornus officinalis* Siebold & Zucc. (16#) herbal medicines (n=3)

| **Heavy metals** | **Calibration curve** | ***r*** | **Repeatability RSD** | **Spike-and-recovery** | **Recovery RSD** | **LOD (µg·L^-1)^** |
| --- | --- | --- | --- | --- | --- | --- |
| **Pb** | *Y*=11148.3*X* +8097.5 | 0.9995 | 12.3% | 97.0% | 0.7% | 3×10^-6^ |
| **Cd** | *Y* =760.5*X*+18.0 | 0.9998 | 13.9% | 104.4% | 3.5% | 5×10^-5^ |
| **As** | *Y*=315.8*X* +510.0 | 0.9997 | 11.9% | 107.8% | 4.3% | 1×10^-3^ |
| **Hg** | *Y* =1700.8*X* +55.0 | 0.9998 | 13.8% | 84.1% | 2.3% | 2×10^-5^ |
| **Cu** | *Y* =750.1*X* +490.5 | 0.9998 | 0.3% | 82.5% | 3.3% | 7×10^-4^ |

### Table S1.16 Methodological verification of ICP-MS for determination of five heavy metals in *Ziziphus jujuba* Mill. Semen (1#) herbal medicines (n=3)

| **Heavy metals** | **Calibration curve** | ***r*** | **Repeatability RSD** | **Spike-and-recovery** | **Recovery RSD** | **LOD (µg·L^-1)^** |
| --- | --- | --- | --- | --- | --- | --- |
| **Pb** | *Y*=11148.3*X* +8097.5 | 0.9995 | 12.3% | 106.1% | 4.8% | 3×10^-6^ |
| **Cd** | *Y* =760.5*X*+18.0 | 0.9998 | 4.4% | 106.1% | 4.1% | 5×10^-5^ |
| **As** | *Y*=315.8*X* +510.0 | 0.9997 | 11.2% | 99.5% | 1.3% | 1×10^-3^ |
| **Hg** | *Y* =1700.8*X* +55.0 | 0.9998 | / | 76.3% | 1.3% | 2×10^-5^ |
| **Cu** | *Y* =750.1*X* +490.5 | 0.9998 | 2.3% | 79.6% | 8.8% | 7×10^-4^ |

* Hg was not detected in this sample

### Table S1.17 Methodological verification of ICP-MS for determination of five heavy metals in *Tetradium ruticarpum* (A.Juss.) T.G.Hartley (8#) herbal medicines (n=3)

| **Heavy metals** | **Calibration curve** | ***r*** | **Repeatability RSD** | **Spike-and-recovery** | **Recovery RSD** | **LOD (µg·L^-1)^** |
| --- | --- | --- | --- | --- | --- | --- |
| **Pb** | *Y*=20110.3*X* +4383.6 | 1.0000 | 2.3% | 109.1% | 9.9% | 6×10^-7^ |
| **Cd** | *Y* =1527.0*X*+64.0 | 1.0000 | 1.7% | 101.4% | 4.4% | 2×10^-5^ |
| **As** | *Y*=668.3*X* +1189.4 | 0.9996 | 5.1% | 110.6% | 3.4% | 3×10^-4^ |
| **Hg** | *Y* =2884.9*X* +66.6 | 0.9999 | 10.5% | 109.1% | 6.4% | 1×10^-5^ |
| **Cu** | *Y* =1554.1*X* +1172.2 | 1.0000 | 0.7% | 87.5% | 3.8% | 1×10^-4^ |

### Table S1.18 Methodological verification of ICP-MS for determination of five heavy metals in *Schisandra chinensis* (Turcz.) Baill. (10#) herbal medicines (n=3)

| **Heavy metals** | **Calibration curve** | ***r*** | **Repeatability RSD** | **Spike-and-recovery** | **Recovery RSD** | **LOD (µg·L^-1)^** |
| --- | --- | --- | --- | --- | --- | --- |
| **Pb** | *Y*=12993.9*X* +5823.1 | 1.0000 | 0.7% | 105.3% | 5.7% | 6×10^-5^ |
| **Cd** | *Y* =908.3*X*+17.4 | 1.0000 | 1.7% | 101.6% | 7.1% | 3×10^-5^ |
| **As** | *Y*=384.2*X* +606.2 | 0.9998 | 7.5% | 104.3% | 1.1% | 8×10^-4^ |
| **Hg** | *Y* =2019.4*X* +207.4 | 0.9999 | 10.5% | 85.5% | 7.8% | 1×10^-5^ |
| **Cu** | *Y* =901.1*X* +463.0 | 1.0000 | 1.8% | 83.6% | 1.6% | 2×10^-4^ |

### Table S1.19 Methodological verification of ICP-MS for determination of five heavy metals in *Houttuynia cordata* Thunb. (2#) herbal medicines (n=3)

| **Heavy metals** | **Calibration curve** | ***r*** | **Repeatability RSD** | **Spike-and-recovery** | **Recovery RSD** | **LOD (µg·L^-1)^** |
| --- | --- | --- | --- | --- | --- | --- |
| **Pb** | *Y*=11148.3*X* +8097.5 | 0.9995 | 1.0% | 107.0%  % | 9.1% | 3×10^-6^ |
| **Cd** | *Y* =760.5*X*+18.0 | 0.9998 | 2.8% | 83.7% | 8.6% | 5×10^-5^ |
| **As** | *Y*=315.8*X* +510.0 | 0.9997 | 2.4% | 105.9% | 8.2% | 1×10^-3^ |
| **Hg** | *Y* =1700.8*X* +55.0 | 0.9998 | 4.4% | 78.6% | 8.4% | 2×10^-5^ |
| **Cu** | *Y* =750.1*X* +490.5 | 0.9998 | 0.8% | 99.5% | 7.8% | 7×10^-4^ |

### Table S1.20 Methodological verification of ICP-MS for determination of five heavy metals in *Gardenia jasminoides*J.Ellis (14#) herbal medicines (n=3)

| **Heavy metals** | **Calibration curve** | ***r*** | **Repeatability RSD** | **Spike-and-recovery** | **Recovery RSD** | **LOD (µg·L^-1)^** |
| --- | --- | --- | --- | --- | --- | --- |
| **Pb** | *Y*=12993.9*X* +5823.1 | 1.0000 | 2.4% | 107.0% | 1.1% | 6×10^-5^ |
| **Cd** | *Y* =908.3*X*+17.4 | 1.0000 | 0.9% | 102.6% | 1.5% | 3×10^-5^ |
| **As** | *Y*=384.2*X* +606.2 | 0.9998 | 6.2% | 108.3% | 1.2% | 8×10^-4^ |
| **Hg** | *Y* =2019.4*X* +207.4 | 0.9999 | 12.6% | 100.4% | 11.6% | 1×10^-5^ |
| **Cu** | *Y* =901.1*X* +463.0 | 1.0000 | 1.9% | 85.7% | 2.4% | 2×10^-4^ |

### Table S1.21 Methodological verification of ICP-MS for determination of five heavy metals in *Citrus × aurantium* L. (4#) herbal medicines (n=3)

| **Heavy metals** | **Calibration curve** | ***r*** | **Repeatability RSD** | **Spike-and-recovery** | **Recovery RSD** | **LOD (µg·L^-1)^** |
| --- | --- | --- | --- | --- | --- | --- |
| **Pb** | *Y*=11148.3*X* +8097.5 | 0.9995 | 9.7% | 109.4% | 1.4% | 3×10^-6^ |
| **Cd** | *Y* =760.5*X*+18.0 | 0.9998 | 7.8% | 101.7% | 3.7% | 5×10^-5^ |
| **As** | *Y*=315.8*X* +510.0 | 0.9997 | 2.9% | 105.0% | 2.3% | 1×10^-3^ |
| **Hg** | *Y* =1700.8*X* +55.0 | 0.9998 | 5.8% | 87.2% | 4.1% | 2×10^-5^ |
| **Cu** | *Y* =750.1*X* +490.5 | 0.9998 | 1.2% | 77.6% | 5.5% | 7×10^-4^ |

### Table S1.22 Methodological verification of ICP-MS for determination of five heavy metals in *Perilla frutescens* (L.) Britton (10#) herbal medicines (n=3)

| **Heavy metals** | **Calibration curve** | ***r*** | **Repeatability RSD** | **Spike-and-recovery** | **Recovery RSD** | **LOD (µg·L^-1)^** |
| --- | --- | --- | --- | --- | --- | --- |
| **Pb** | *Y*=11509.6*X* +9428.9 | 0.9995 | 1.8% | 103.0% | 8.8% | 3×10^-6^ |
| **Cd** | *Y* =9428.9*X*+26.0 | 1.0000 | 4.8% | 100.0% | 1.5% | 5×10^-5^ |
| **As** | *Y*=317.9*X* +498.7 | 0.9996 | 8.1% | 103.1% | 5.7% | 1×10^-3^ |
| **Hg** | *Y* =1665.6*X* +239.0 | 0.9999 | 2.0% | 81.7 | 9.4% | 2×10^-5^ |
| **Cu** | *Y* =760.2*X* +758.7 | 1.0000 | 1.8% | 88.6% | 1.6% | 7×10^-4^ |

### Table S1.23 Methodological verification of ICP-MS for determination of five heavy metals in *Plantago asiatica* L. (4#) herbal medicines (n=3)

| **Heavy metals** | **Calibration curve** | ***r*** | **Repeatability RSD** | **Spike-and-recovery** | **Recovery RSD** | **LOD (µg·L^-1)^** |
| --- | --- | --- | --- | --- | --- | --- |
| **Pb** | *Y* =12882.4*X* +3399.7 | 0.9999 | 3.2% | 92.1% | 8.8% | 2×10^-6^ |
| **Cd** | *Y*= 1111.0*X* +21.4 | 0.9999 | 1.5% | 94.1% | 8.6% | 2×10^-5^ |
| **As** | *Y* =510.6*X* +936.5 | 0.9999 | 7.7% | 97.2% | 7.6% | 5×10^-4^ |
| **Hg** | *Y* =1900.9*X* + 50.0 | 0.9999 | 8.0% | 98.3% | 8.6% | 4×10^-5^ |
| **Cu** | *Y*= 1181.71*X* + 6461.5 | 0.9999 | 6.7% | 80.9% | 8.8% | 3×10^-4^ |

### Table S2 Permissible limits of five heavy metals in medicinal herbs (products) from 27 countries and international organizations

| **No.** | **Published by** | **Published for** | **Limits of five heavy metals (mg·kg^-1^)** | | | | | **References** |
| --- | --- | --- | --- | --- | --- | --- | --- | --- |
|  |  |  | **Lead (Pb)** | **Arsenic (As)** | **Cadmium (Cd)** | **Mercury (Hg)** | **Copper (Cu)** |  |
| 1 | WHO 2007 | Crude herbal drugs | 10.0 | 1.0 | 0.3 | -- | -- | WHO Guidelines for Assessing Quality of Herbal Medicines with Reference to Contaminants and Residues |
| 2 | ISO | Chinese herbal medicine | 10.0 | 4.0 | 2.0 | 3.0 | -- | ISO/TC249, 2015 |
| 3 | European Union | Herbal medicine | 5.0 | -- | 0.5 | 0.3 | -- | EU Pharmacopeia, EP 9.2, 2014 |
| 4 | NSF International 2008 | Dietary supplements | 10.0 | 5.0 | 0.3 | 0.2 | -- | NSF International Draft Standard 173-2008 |
| 5 | Ph. Eur. Monograph 2008 | Herbal medicine | 5.0 | -- | 0.5 | 0.1 | -- | O. Awodele et al., 2008 |
| 6 | Regulation (EC) 629/2008 Commission Regulation (2008) | Herbal medicine | 3.0 | -- | 1.0 | 0.1 | -- | O. Aw odele et al., 2008 |
| 7 | Chinese Pharmacopeia (2020) | Chinese herbal medicine | 5.0 | 2.0 | 1.0 | 0.2 | 20.0 | Chinese Pharmacopeia (2020) |
| 8 | Green standards of medicinal plants and preparations for foreign trade and economy (China) | Herbal medicine | 5.0 | 2.0 | 0.3 | 0.2 | -- | WM/T2-2004 |
| 9 | Hong Kong, China | Chinese herbal medicine | 5.0 | 2.0 | 1.0 | 0.2 | 150.0 | Vol 1, Hong Kong Chinese Materia Medica Standard, 2005 |
| 10 | Macau, China | Raw medicinal herbs and CHM for external use | 20.0 | 5.0 | -- | 0.5 | 150.0 | Macau Technique Directive 02-2003 |
| 11 | Taiwan, China | Herbal products | 10.0 | 3.0 | 0.5 | 0.5 | -- | Regulations for Registration of Medicinal Products |
| 12 | Japan | Raw medicinal herbs | 20.0 | 5.0 | -- | -- | 10.0 | Karimi et al., 2008 |
| 13 | Singapore | Chinese proprietary Medicines, traditional medicines and raw medicinal herbs | 20.0 | 5.0 | -- | 0.5 | -- | Health Sciences Authority |
| 14 | India | Herbal medicine | 10.0 | 3.0 | 0.3 | 1.0 | -- | AYUSH, 2005 |
| 15 | South Korea | Raw medicinal herbs | 5.0 | 3.0 | 0.3 | 0.2 | -- | Announcement of Drug Safety Office, South Korea, 2005-62 |
| 16 | North Korea | Herbal materials | 30.0 | -- | -- | -- | -- | R.A.Street, 2012 |
| 17 | Malaysia | Finished herbal products | 10.0 | 5.0 | 0.3 | 0.5 | -- | R.A Street, 2012 |
| 18 | Thailand | Herbal materials | 10.0 | 4.0 | 0.3 | -- | -- | Karimi et al., 2008 |
| 19 | Vietnam | Herbs | 10.0 | 4.0 | 1.0 | 0.5 | -- | Pharmacopoeia of Vietnam |
| 20 | Germany and France | Products of plant origin | 5.0 | 5.0 | 0.2 | 0.1 | -- | Gasser et al., 2009 |
| 21 | Italy | Crude herbal drugs | 3.0 | -- | 0.5 | 0.3 | -- | Italian Pharmacopeia (FUI),2002 |
| 22 | U.K | Herbal medicine | 5.0 | 5.0 | 1.0 | 0.1 | -- | Harris et al., 2011 |
| 23 | U.S.A | Herbal extracts | 5.0 | 2.0 | 0.3 | 0.2 | -- | U.S. Pharmacopeia, USP40-NF35, 2017 |
| 24 | U.S.A-California | Herbs | 0.5 | 10.0 | 4.1 | -- | -- | State of California Proposition 65 |
| 25 | Canada | Raw herbal materials | 10.0 | 5.0 | 0.3 | 0.2 | -- | Street et al., 2006 |
| 26 | Brazil | Herbal Tea | -- | 0.6 | 0.4 | -- | -- | Anvisa, 2013 |
| 27 | Australia | Herbal medicine | 5.0 | -- | 1.0 | 0.1 | -- | Shiv B et al., 2017 |
| Minimum MRLs from different regions | | | 0.5 | 0.6 | 0.2 | 0.02 | 10.0 |  |
| Range | | | 3.0-30.0 | 0.6-5.0 | 0.2-4.0 | 0.02-1.0 | 10.0-150.0 |  |

### Table S3 Over-limit ratio (%) of five heavy metals in 32 producing areas

| No. | Producing area | Total number of samples | Total number of over-limit samples | Total over-limit ratio (%) | Cu  (%) | As  (%) | Cd  (%) | Hg  (%) | Pb  (%) |
| --- | --- | --- | --- | --- | --- | --- | --- | --- | --- |
| 1 | Chongqing | 13 | 10 | 76.92 | 7.69 | 0.00 | 0.77 | 0.00 | 7.69 |
| 2 | Guizhou | 16 | 9 | 56.25 | 0.00 | 6.25 | 0.56 | 6.25 | 6.25 |
| 3 | Hunan | 91 | 48 | 52.75 | 0.00 | 2.20 | 0.48 | 9.89 | 5.49 |
| 4 | Guangxi | 130 | 68 | 52.31 | 3.08 | 4.62 | 0.48 | 2.31 | 13.85 |
| 5 | Fujian | 16 | 8 | 50.00 | 0.00 | 6.25 | 0.38 | 6.25 | 25.00 |
| 6 | Vietnam | 2 | 1 | 50.00 | 0.00 | 0.00 | 0.50 | 0.00 | 0.00 |
| 7 | Sichuan | 144 | 69 | 47.92 | 1.39 | 4.17 | 0.43 | 1.39 | 10.42 |
| 8 | Guangdong | 38 | 18 | 47.37 | 0.00 | 0.00 | 0.47 | 0.00 | 10.53 |
| 9 | Hubei | 196 | 85 | 43.37 | 5.10 | 7.14 | 0.35 | 7.14 | 7.65 |
| 10 | Anhui | 125 | 43 | 34.40 | 0.00 | 2.40 | 0.28 | 7.20 | 0.00 |
| 11 | Hainan | 3 | 1 | 33.33 | 0.00 | 0.00 | 0.33 | 0.00 | 0.00 |
| 12 | Guangdong | 71 | 22 | 30.99 | 1.41 | 2.82 | 0.23 | 5.63 | 4.23 |
| 13 | Gansu | 94 | 25 | 26.60 | 3.19 | 20.21 | 0.04 | 0.00 | 3.19 |
| 14 | Hebei | 94 | 24 | 25.53 | 0.00 | 1.06 | 0.15 | 4.26 | 8.51 |
| 15 | Zhejiang | 42 | 10 | 23.81 | 2.38 | 0.00 | 0.10 | 7.14 | 4.76 |
| 16 | Henan | 201 | 45 | 22.39 | 2.99 | 2.49 | 0.14 | 2.99 | 4.98 |
| 17 | Heilongjiang | 9 | 2 | 22.22 | 0.00 | 0.00 | 0.22 | 0.00 | 0.00 |
| 18 | Yunnan | 36 | 7 | 19.44 | 0.00 | 8.33 | 0.19 | 0.00 | 8.33 |
| 19 | Jiangxi | 128 | 23 | 17.97 | 0.78 | 2.34 | 0.09 | 6.25 | 2.34 |
| 20 | Qinghai | 19 | 3 | 15.79 | 0.00 | 10.53 | 0.16 | 5.26 | 10.53 |
| 21 | Shanxi | 85 | 10 | 11.76 | 1.18 | 3.53 | 0.06 | 1.18 | 4.71 |
| 22 | Inner Mongolia | 18 | 2 | 11.11 | 0.00 | 5.56 | 0.06 | 0.00 | 5.56 |
| 23 | Jiangsu | 18 | 1 | 5.56 | 0.00 | 0.00 | 0.06 | 0.00 | 0.00 |
| 24 | Sinking | 56 | 3 | 5.36 | 1.79 | 3.57 | 0.00 | 0.00 | 0.00 |
| 25 | Liaoning | 39 | 2 | 5.13 | 0.00 | 0.00 | 0.00 | 5.13 | 0.00 |
| 26 | Shaanxi | 22 | 1 | 4.55 | 0.00 | 0.00 | 0.05 | 0.00 | 0.00 |
| 27 | Ningxia | 30 | 1 | 3.33 | 0.00 | 0.00 | 0.03 | 0.00 | 0.00 |
| 28 | Beijing | 10 | 0 | 0.00 | 0.00 | 0.00 | 0.00 | 0.00 | 0.00 |
| 29 | Jilin | 23 | 0 | 0.00 | 0.00 | 0.00 | 0.00 | 0.00 | 0.00 |
| 30 | Burma | 1 | 0 | 0.00 | 0.00 | 0.00 | 0.00 | 0.00 | 0.00 |
| 31 | Nepal | 2 | 0 | 0.00 | 0.00 | 0.00 | 0.00 | 0.00 | 0.00 |
| 32 | Thailand | 1 | 0 | 0.00 | 0.00 | 0.00 | 0.00 | 0.00 | 0.00 |
| Total | | 1773 | 541 | 30.51 | 1.75 | 4.17 | 0.23 | 3.84 | 5.75 |

### Table S4 Chronic and acute adverse effects of five heavy metals on health

| Metal | Affected organs or systems | Chronic or acute | Symptoms and syndromes | References |
| --- | --- | --- | --- | --- |
| Cd (cadmium) | Renal, prostate, skeletal, pulmonary, nervous, liver, vascular, immune, endocrine, and cardiovascular systems, brain | Chronic | Prostatic proliferative lesions, proteinuria, glucosuria, aminoaciduria, renal tubular dysfunction, irreversible impairment of the renal tract, renal failure;  osteoporosis, osteomalacia, bone fractures, hypertension;  DNA strand damage, DNA protein cross-links; potent cell poison, cell death or increase in cell proliferation; protein damage and subsequently neurodegeneration, peripheral neuropathy,  Hematological and immunological effects, anemia, liver lesions;  cataract formation in the eyes | Sawut R et al., Maiga A et al., Aziz MA et al., Mulaudzi RB et al., Khan MA et al., Sandilyan S et al., Mahurpawar M et al. |
|  |  | Acute | Increased oxidative stress, membrane damage and loss of membrane-bound enzymes like ATPases;  nausea, vomiting, abdominal cramps, dyspnea and muscular weakness;  anosmia, cerebrovascular infarction;  pulmonary odema , emphysema, bronchiolitis, alveolitis, and death |  |
|  |  | Cancer | Human carcinogen (group I, by IARC); prostate, kidney, lung, and cardiac failure; cancers |  |
| Pb (lead) | Endocrine, vascular, nervous, immune, hematopoietic, renal, skeletal, muscular, reproductive, and cardiovascular liver, and immune systems, placenta | Chronic | Diabetes mellitus, cardiac disorders;  Poor muscle coordination, muscle and joint pain, a decrease handgrip strength, paralysis of the wrist joint;  damage to the gastrointestinal tract (GIT) and urinary tract resulting in bloody urine, convulsions and chronic nephritis of the kidneys;  permanent brain damage, high blood pressure, hearing and vision impairments;  inhibition of the synthesis of haemoglobin; cardiovascular system and acute and chronic damage to the central nervous system (CNS) and peripheral nervous system (PNS) with reduced nerve conduction velocity and reduced dermal sensibility;  Fertility, increase chances of miscarriage or birth defects, teratogenic symptoms, | Sawut R et al., Maiga A  Et al., Mulaudzi RB et al., Khan MA et al., Jarup L et al., Mahurpawar M et al. |
|  |  | Acute | headache, irritability, abdominal pain and various symptoms related to the nervous system, fatigue, nausea, vomiting and gastrointestinal problems and anoxia, colic, anemia, coma, delirium and death;  Encephalopathy, sleeplessness and restlessness;  Children with behavioral disturbances, developmental delays, learning and concentration difficulties, poor intelligence quotient (IQ), memory deterioration, prolonged reaction time and reduced ability to understand;  acute psychosis, confusion and reduced consciousness;  proximal renal tubular damage;  developing nervous system systems, congenial heart abnormalities;  It crosses the placenta barrier and can accumulate in breast milk, leading to spontaneous abortions and chromosome aberrations |  |
|  | | Cancer | possible human carcinogen (by IARC); lung and stomach cancer, and gliomas |  |
| Cu (Cooper) | Nervous, respiratory, digestive systems, DNA, and skin |  | Breaks of DNA strands, damage to membranes and mitochondria;  Alzheimer’s disease, Prion disease, Wilsons’s disease, and amylotrophic lateral sclerosis;  dermatitis, irritation of the upper respiratory tract, fever;  gastro-intestinal problems, abdominal pain, nausea, metallic taste in the mouth, diarrhea, vomiting, dizziness, hepatic cirrhosis, intelligence;  severe mucosal irritation and corrosion, widespread capillary damage, central nervous system irritation, depression;  hepatic and renal damage, and even death; | Maiga A et al., Aziz MA et al., Mulaudzi RB et al., Mahurpawar M et al. |
| As (Arsenic) | Pulmonary, nervous systems, skin | Chronic | Decreased production of red and white blood cells, abnormal heart rhythm, hypertension, diabetes, cardiovascular disease, and neurodegenerative disorders, perforation of nasal septum;  reproductive effects;  skin lesions, such as hyperkeratosis and pigmentation changes, dermatomes;  permanent damage to the brain and kidneys, and death  acts to coagulate protein, forms complexes with coenzymes and inhibits the production of adenosine triphosphate (ATP) during respiration.  presents a disorder similar to, and often confused with Guillain-Barre syndrome, a nerve inflammation that causes muscle weakness | Mulaudzi RB et al., Jiwan S et al., Mahurpawar M et al. |
|  |  | Acute | gastrointestinal symptoms;  bone marrow depression, haemolysis, hepatomegaly, melanosis, polyneuropathy and encephalopathy;  peripheral vascular disease, gangrenous changes;  severe disturbances of the cardiovascular and central nervous systems, and eventually death.  Developing nervous system, renal failure, congenial heart abnormalities |  |
|  |  | Cancer | Bladder, kidney, respiratory, and skin cancers, possibly carcinogenic in compounds of all its oxidation states and high-level exposure can cause death. |  |
| Hg (mercury) | Nervous, renal and respiratory systems | Acute | Lung damage | Jarup L et al., Mahurpawar M et al. |
|  |  | Chronic | Psychological symptoms such as tremor, changes in personality, restlessness, anxiety, sleep disturbance and depression;  Impaired neurodevelopment in children leading to learning difficulties, poor memory and shortened attention spans.  Cerebral palsy among children, irregular pulse rate, tachycardia, excitement, psychic disorders, gingivities, headache, dizziness, insomnia;  Proteinuria, male fertility. |  |

### Fig.S1. The over-limit Estimated Daily Intakes (EDI) of four heavy metals in herbal medicines.

1.

All scores were calculated with maximal concentrations of each herbal medicine. Only EDIs above their corresponding provisional tolerable daily intakes (PTDI) were shown here. (The PTDIs (mg·kg^-1^·day^-1^) of As, Hg, Pb, and Cd are 0.00214, 0.00057, 0.00357, and 0.00083, respectively).

### Fig.S2. The over-limit Hazard Quotients (HQ) of four heavy metals in 21 herbal medicines.



 All scores were calculated with maximal concentrations of each herbal medicine. Only HQs above one were shown here.

### Fig.S3.1 Spearman correlation coefficient of metal relations in flos


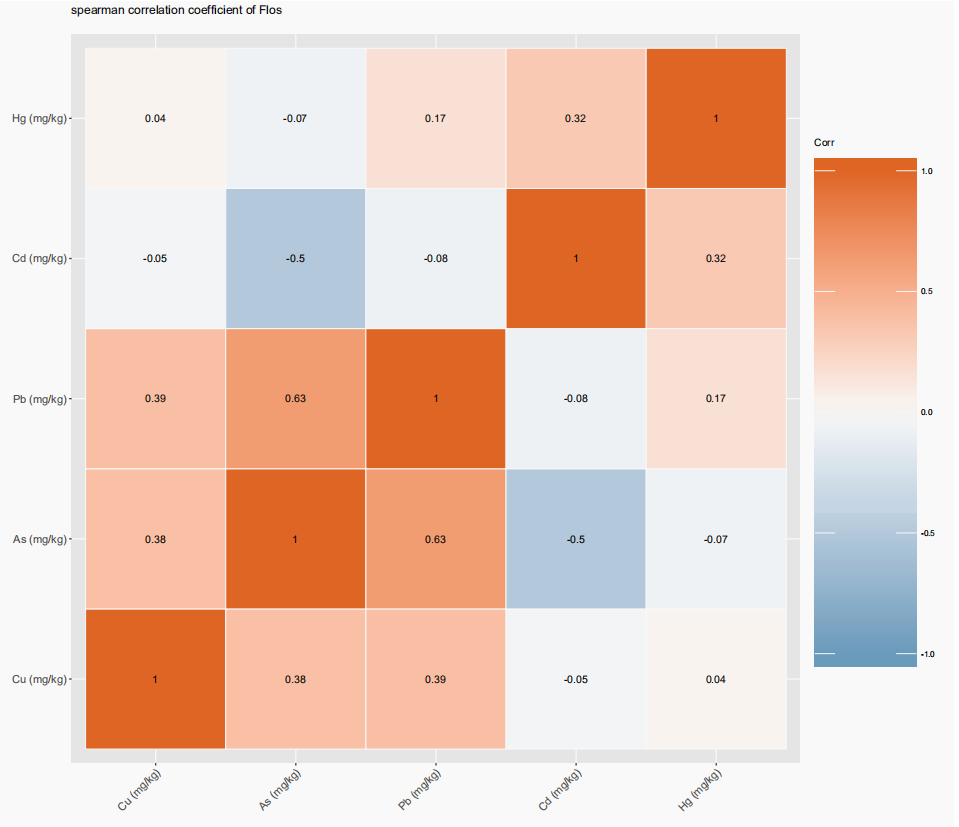


### Fig.S3.2 Spearman correlation coefficient of metal relations in folium & cortex


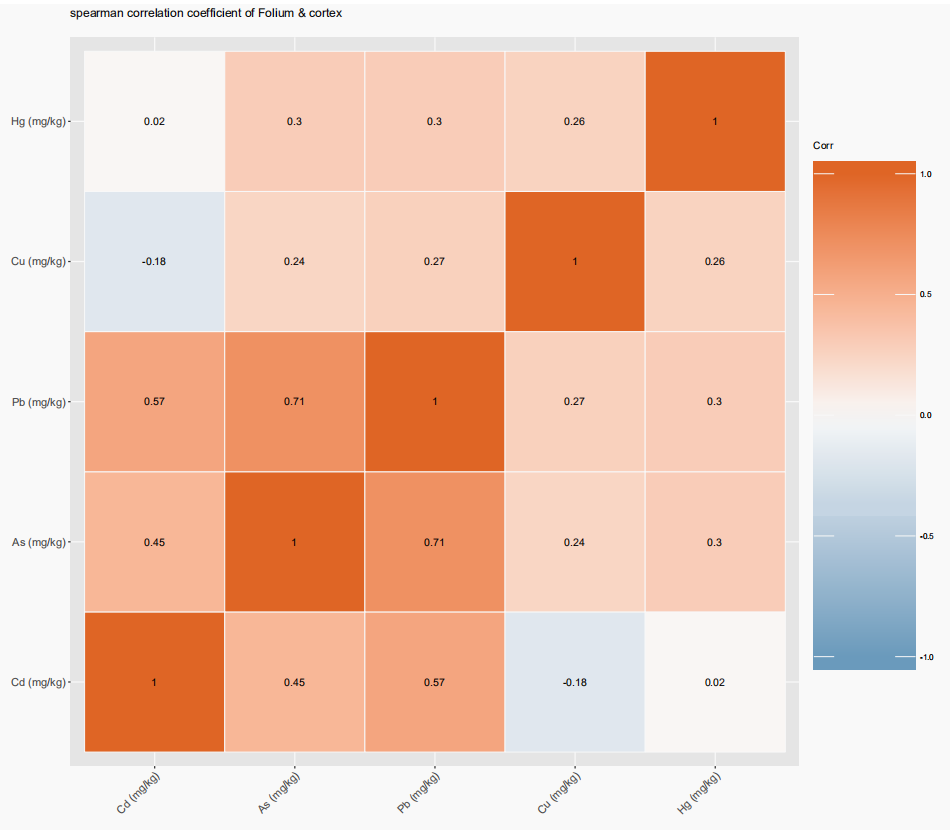


### Fig.S3.3 Spearman correlation coefficient of metal relations in fructus & semen


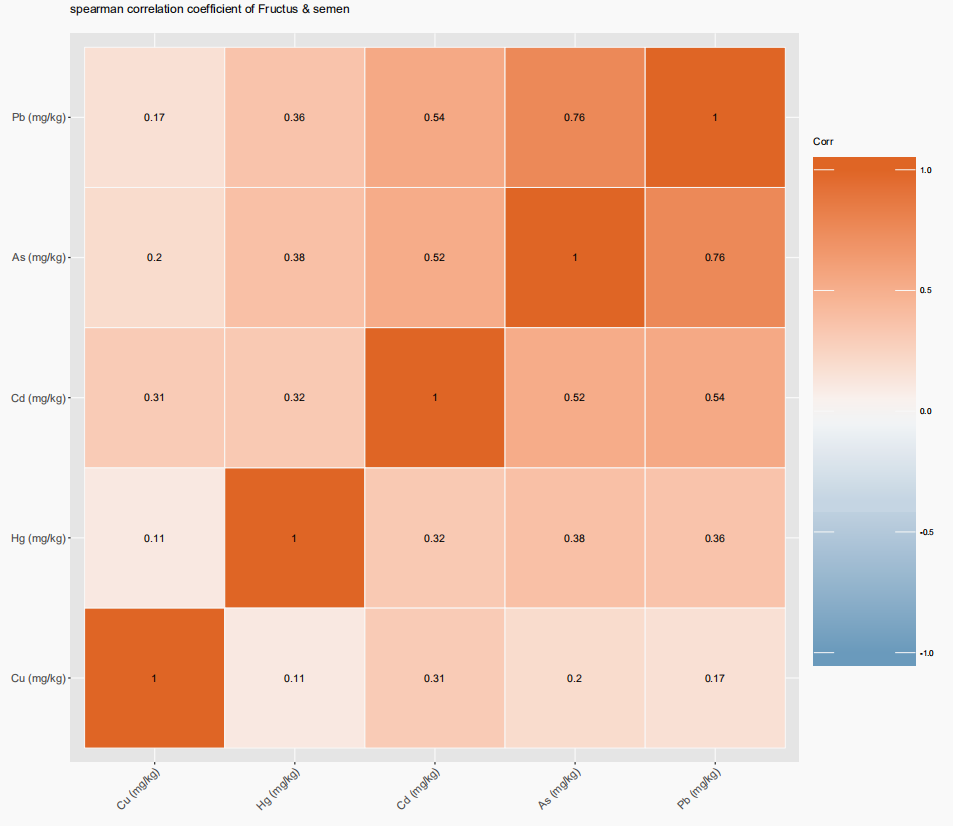


### Fig.S3.4 Spearman correlation coefficient of metal relations in herba & others


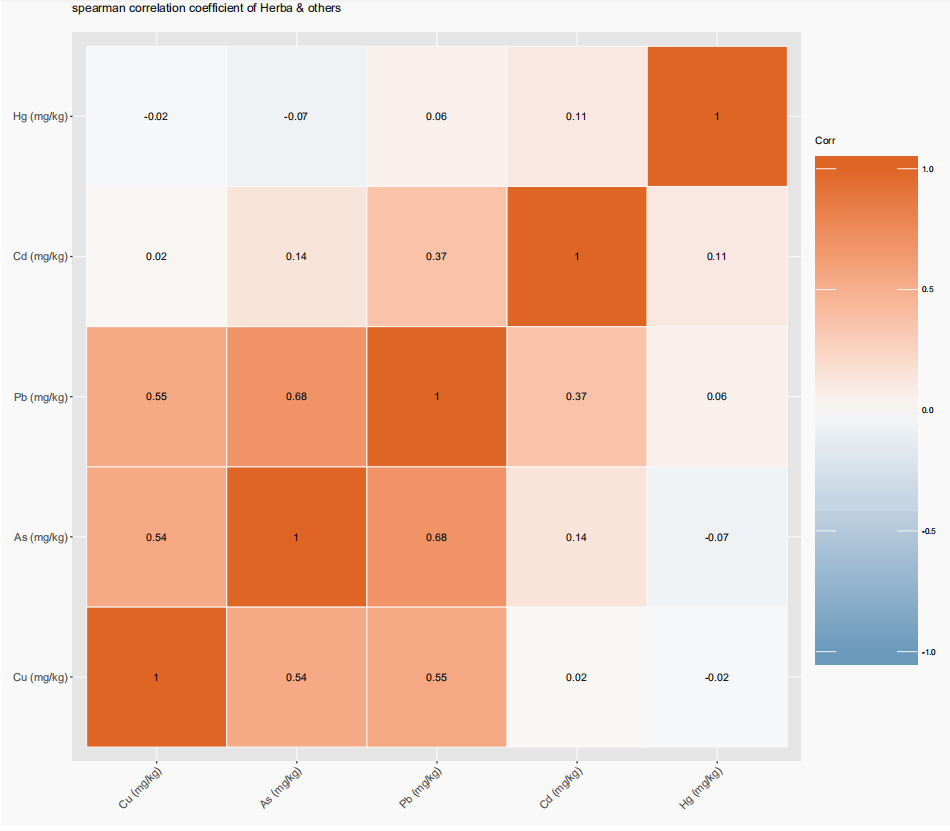


### Fig.S3.5 Spearman correlation coefficient of metal relations in radix & rhizoma


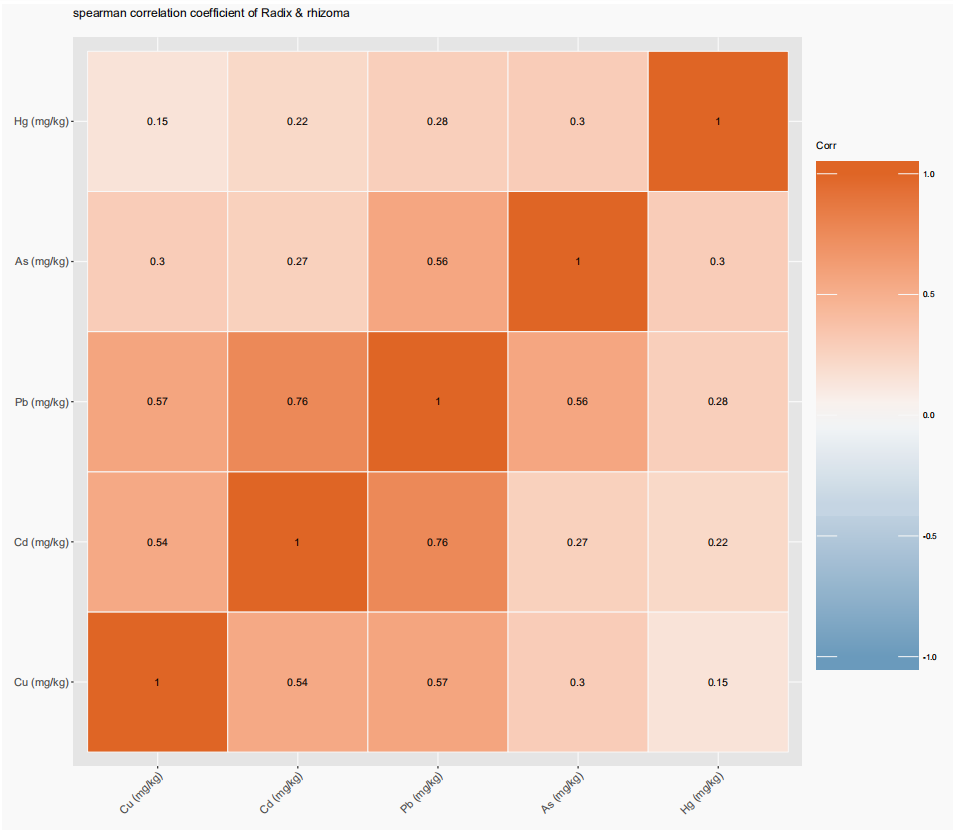


### Fig.S4.1 Principle Components Analysis (PCA) of five heavy metal contents in five plant properties

**
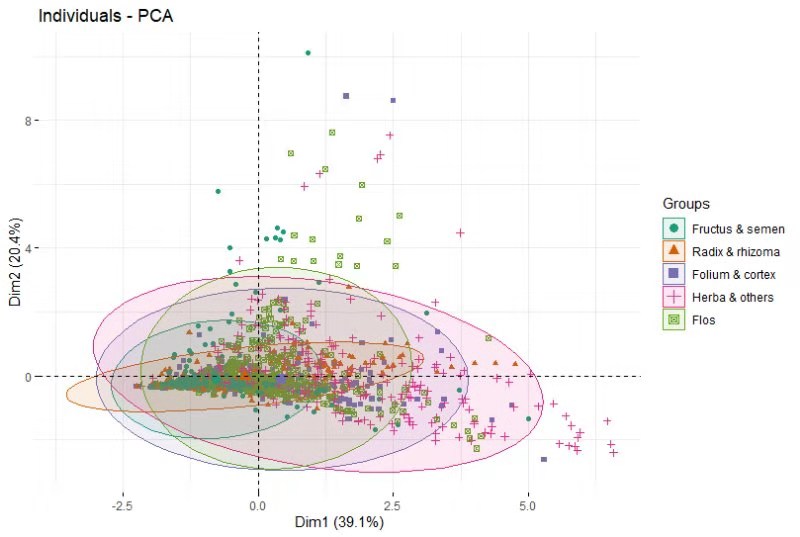
**

### Fig.S4.2 PCA of five heavy metals in 32 producing areas


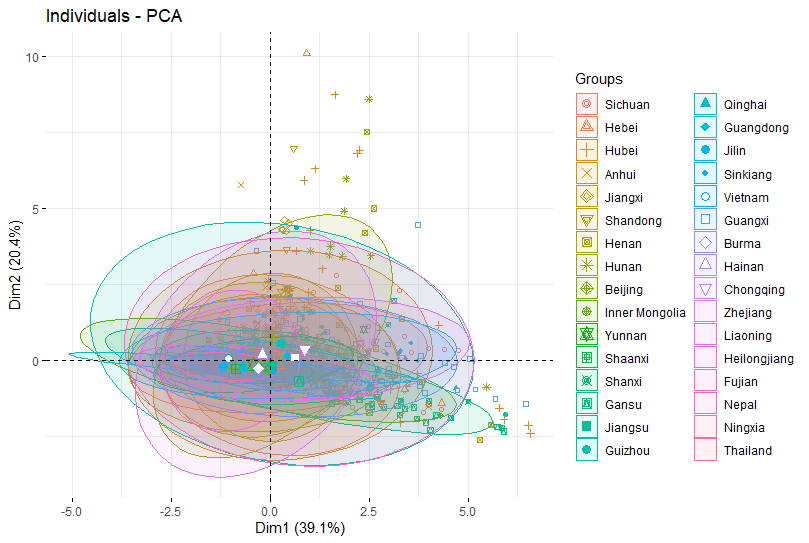


### Fig.S5 Analysis of Similarities (ANOSIM) of five heavy metal contents in five medicinal plant properties

**
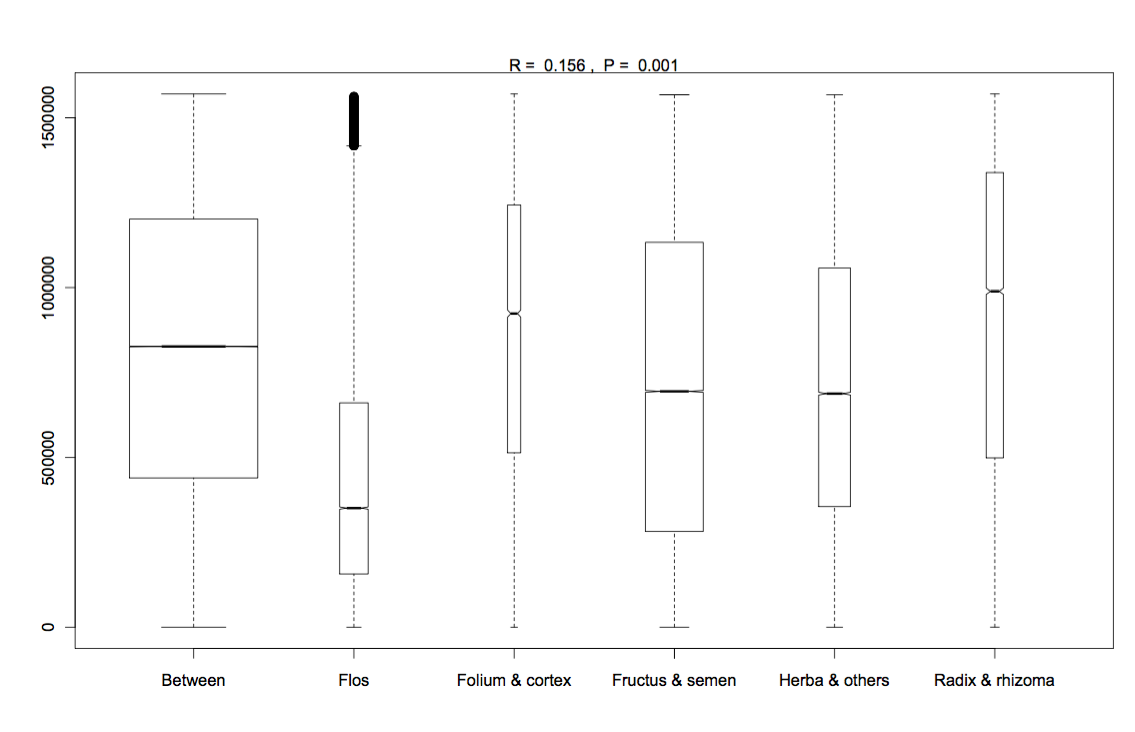
**
